# Trophic Transition Enhanced Biomass and Lipid Production of the Unicellular Green Alga *Scenedesmus acuminatus*

**DOI:** 10.3389/fbioe.2021.638726

**Published:** 2021-05-21

**Authors:** Hu Zhang, Liang Zhao, Yi Chen, Mianmian Zhu, Quan Xu, Mingcan Wu, Danxiang Han, Qiang Hu

**Affiliations:** ^1^Center for Microalgal Biotechnology and Biofuels, Institute of Hydrobiology, Chinese Academy of Sciences, Wuhan, China; ^2^College of Life Sciences, University of Chinese Academy of Sciences, Beijing, China; ^3^Key Laboratory for Algal Biology, Institute of Hydrobiology, Chinese Academy of Sciences, Wuhan, China; ^4^The Innovative Academy of Seed Design, Chinese Academy of Sciences, Beijing, China; ^5^Institute for Advanced Study, Shenzhen University, Shenzhen, China; ^6^State Key Laboratory of Freshwater Ecology and Biotechnology, Institute of Hydrobiology, Chinese Academy of Sciences, Wuhan, China

**Keywords:** *Scenedesmus acuminatus*, heterotrophically grown cells, lipid production, transcriptomic analysis, stress responses

## Abstract

Microalgal heterotrophic cultivation is an emerging technology that can enable producing high cell-density algal cell cultures, which can be coupled with photoautotrophic cultivation for valuable chemicals such as lipids manufacturing. However, how the heterotrophically grown algal cells respond to the lipid-inducing conditions has not been fully elucidated so far. In this study, when the heterotrophically grown *Scenedesmus acuminatus* cells were subjected to the high light (HL) and nitrogen-limited (NL) conditions, both the biomass and lipid productivity were enhanced as compared to that of the photoautotrophically grown counterparts. The chlorophyll *a* fluorometry analysis showed that the Fv/Fm and Y(II) of the heterotrophically grown cells subjected to the HL and NL conditions was recovered to the maximum value of 0.75 and 0.43, respectively, much higher than those of the photoautotrophically grown cells under the same stress conditions. Transcriptomic analysis revealed that heterotrophically grown cells fully expressed the genes coding for the photosystems proteins, including the key photoprotective proteins D1, PsbS, light-harvesting-complex (LHC) I and LHC II. Meanwhile, downregulation of the carotenoid biosynthesis and upregulation of the glycolysis/gluconeogenesis, tricarboxylic acid (TCA) cycle and oxidative phosphorylation pathways were observed when the heterotrophically grown cells were subjected to the HL and N-limited conditions for lipid production. It was deduced that regulation of these pathways not only enhanced the light utilization but also provided the reducing power and ATP by which the biomass accumulation was significantly elevated. Besides, upregulation of the acetyl-CoA carboxylase/biotin carboxylase, digalactosyl diacylglycerol synthase and diacylglycerol acyltransferase 2 encoding genes may be attributable to the enhanced lipid production. Understanding the cellular responses during the trophic transition process could guide improvement of the strength of trophic transition enhancing microalgal biomass and lipid production.

## Introduction

Microalgae are promising feedstocks for liquid biofuels production because of their high photosynthetic efficiencies, fast growth rates, high lipid contents and no competition with food production for arable land during cultivation (Yin et al., 2020). Production of biofuels from photoautotrophically grown microalgal biomass has already proved to be technically feasible at both lab and pilot-scales (Sajjadi et al., 2018; Yang et al., 2018; Tang et al., 2020). However, commercialization of microalgae-derived biofuels has been limited by the high costs, largely associated with biomass production (Su et al., 2017; Aziz et al., 2020). In the past decades, various cultivation strategies besides photoautotrophic cultivation have been emerging to enhance microalgal lipid production so as to balance the costs, which include heterotrophic cultivation, photoautotrophy-to-heterotrophy cultivation and heterotrophy-to-photoautotrophy cultivation (Xiong et al., 2010; Han et al., 2012; Zheng et al., 2013).

Among these cultivation modes, the heterotrophic cultivation mode offers many advantages that can enable fast growth and high biomass productivity for microalgae (Chen et al., 2020), while photoautotrophic cultivation is essential for producing many compounds of which the synthesis are highly dependent on photosynthesis, such as lipids requiring large amounts of photosynthetically produced reducing equivalent and fatty acids (Li-Beisson et al., 2015). Accordingly, heterotrophy-to-photoautotrophy cultivation takes advantages of both the cultivation modes, which are producing high-quality biomass via heterotrophy and inducing biosynthesis of particular end-products under photoautotrophic conditions. Currently, such a cultivation mode has been adopted for many algal strains for the manufacturing of a wide spectrum of chemicals, which included, but are not restricted to, *Chlorella* spp. and *Scenedesmus acuminatu*s for lipid production (Han et al., 2012; Jin et al., 2020), *Haematococcus pluvialis* and *Chromochloris zofingiensis* for astaxanthin production (Wan et al., 2015; Sun et al., 2019), and *Botryococcus braunii* for hydrocarbons production (Wan et al., 2019). It is noteworthy that when the coupled cultivation mode was utilized for *Chlorella* spp. and *S. acuminatu*s, the biomass yield and lipid productivity were significantly enhanced as compared with traditional photoautotrophic cultivation (Han et al., 2012; Jin et al., 2020).

Due to the above-mentioned merits of the heterotrophy-to-photoautotrophy cultivation mode, several studies have already been conducted to dissect the physiological and biochemical changes during the trophic transition of *Chlorella pyrenoidosa* and *Chromochloris zofingiensis* (Fan et al., 2015; Roth et al., 2019). However, the biological mechanisms underlying the enhanced microalgal biomass and lipid production under the heterotrophy-to-photoautotrophy cultivation mode has not been fully elucidated so far, especially for how heterotrophically grown algal cells adapt to the lipid-inducing conditions.

*Scenedesmus acuminatus* is a freshwater green alga capable of accumulating lipids as high as up to 50% of the dry wight under high light (HL) and nitrogen-limited (NL) conditions in photoautotrophic cultivation (Zhang et al., 2018). The alga is also able to grow robustly under heterotrophic conditions, reaching an ultrahigh-cell-density of 286 g L^–1^ (Jin et al., 2020). Interestingly, both the biomass concentration and lipid content of *S. acuminatus* cells were much higher under the heterotrophy-to-photoautotrophy cultivation conditions than those under the traditional photoautotrophic cultivation conditions (Jin et al., 2020). Thus, this alga is an ideal model to investigate the impact of the trophic transition from heterotrophy to photoautotrophy on algal cells. In this study, the heterotrophically grown cells (HC) of *S. acuminatus* and photoautotrophically grown ones (PC) were both subjected to the HL and NL conditions, and the cellular growth, lipid content and photosynthetic efficiency was compared between them, respectively. The responses of the genes involved in photosynthesis, central carbon metabolism, carotenoid biosynthesis and fatty acids biosynthesis were also surveyed by RNA-seq analysis in order to advance our understanding about mechanisms underlying the enhanced biomass and lipid production of *S. acuminatus* during trophic transition.

## Materials and Methods

### Algal Strains and Culturing Conditions

The green alga *S. acuminatus* was isolated from South Lake of Guangzhou, China (Jin et al., 2020). For heterotrophic cultivation, algae cells were maintained in the modified Endo growth medium, containing glucose 30 g L^–1^, KNO_3_ 3 g L^–1^, KH_2_PO_4_ 1.2 g L^–1^, MgSO_4_⋅7H_2_O 1.2 g L^–1^, trisodium citrate 0.2 g L^–1^, FeSO_4_⋅7H_2_O 0.016 g L^–1^, EDTA-Na_2_ 2.1 mg L^–1^, CaCl_2_⋅2H_2_O 0.03 g L^–1^, H_3_BO_3_ 2.86 mg L^–1^, ZnSO_4_⋅7H_2_O 0.222 mg L^–1^, MnCl_2_⋅4H_2_O 1.81 mg L^–1^, Na_2_MoO_4_ 0.021 mg L^–1^, CuSO_4_⋅2H_2_O 0.07 mg L^–1^. Heterotrophic cultivation was performed as described in the previous study (Jin et al., 2020). Briefly, the algal cells cultured in 1 L Erlenmeyer flask containing 300 mL medium on a rotary shaker at 180 rpm in dark was used as inoculum for the heterotrophic cultivation in the 7.5-L fermenter (BIOFLO and CELLIGEN 310, New Brunswick, United States). The initial culture volume of fermenter was 2.8 L, and the airflow rate was 2.8 L min^–1^. Dissolved oxygen was controlled automatically above 40% (*v*/*v*) via being coupled with the stirring speed. The initial glucose and urea concentration were 5 and 0.337 g L^–1^, respectively, corresponding to the C/N ratio of 12. The feeding medium used during fermentation process was the 25-fold concentrated growth medium used for batch culturing, containing 750 g L^–1^ of glucose. Stepwise constant feeding strategy was adopted to control the glucose concentration below 5 g L^–1^ during the cultivation. Besides, the temperature and pH was set at 30°C and 6.0, respectively. Heterotrophically grown algae cells were used as inoculum for lipid induction when cell biomass reached *ca.* 200 g L^–1^ after 144 h of fermentation.

The BG-11 growth medium was used for photoautotrophic cultivation of *S. acuminatus* cells (Rippka et al., 1979). The algal cells were cultured in 800 mL column photobioreactors (PBRs) (i.d. 5 cm) containing 750 mL of BG-11 growth medium, and grown to exponential phase (4–5 days) under continuous light intensity of 90 μmol m^–2^ s^–1^ at 25 ± 2.5°C. Mixing and aeration were provided by bubbling air containing 2.0% (*v/v*) CO_2_ with a flow rate of 0.2 vvm.

The HC and PC were harvested by centrifugation (3000 *g*, 5 min) and resuspended in the NL BG-11 growth medium with 0.09375 g L^–1^ NaNO_3_, corresponding to 1/16 of the original nitrate concentration of BG-11. The initial chlorophyll concentrations of above two cultures were adjusted to *ca.* 6.0 mg L^–1^. The cell cultures were subjected to the continuously HL intensity of 400 μmol m^–2^ s^–1^ at 25 ± 2.5°C to induce lipid production.

### Growth and Lipid Contents Determination

Cell growth was estimated by measuring the cellular dry weight (DW), cell number and chlorophyll concentration of the algal cell cultures. DW was measured according to the method described by Wen et al. (2016). Cell numbers were counted by using a hemacytometer (Improved Neubauer, United States) under the microscope (BX51, Olympus, Japan). Chlorophyll and carotenoids contents were determined by using the previously described method (Ma et al., 2017). Briefly, an aliquot (10 mL) of culture suspension was centrifuged (4000 × *g*, 10 min, 4°C) to collect the cell pellets. After discarding the supernatants, the cell pellets were kept at −80°C for 24 h. Methanol (10 mL) was added to the centrifuge tube, which was then placed in a water-bath at 75°C for 20 min in dark, followed by centrifugation (4000 × *g*, 10 min, 4°C). The supernatants were transferred to a cuvette for measurement of optical density at 653 nm (OD_653_), 666 nm (OD_666_) and 470 nm (OD_470_).

C:aChlorophyllaconcentration=(15.65×OD666-7.34×OD)653×VMeOH/Valgae

C:bChlorophyllbconcentration=(27.05×OD653-11.21×OD)666×VMeOH/Valgae

Chlorophyllconcentration=Chlorophyllaconcentration+Chlorophyll⁢b⁢concentration

Carotenoidsconcentration=(1000×A-4702.86×C-a129.2×C)b/221

where V_*MeOH*_ is the volume of methanol and V_*algae*_ is the volume of microalgae suspension used for the extraction of pigments.

The contents of fatty acid methyl esters (FAMEs) were determined according to the method described in our previous study (Jia et al., 2015). Briefly, 25 μL of the 10 mg mL^–1^ methyl tridecanoate, 200 μL of the chloroform:methanol (2:1, v/v) and 300 μL of the 5% (v/v) HCl:methanol were added to 10 mg sample and was transesterified in tightly sealed vials at 85°C for 1 h. FAMEs were extracted with 1 mL of hexane at room temperature for 1 h. Then the extracted FAMEs with pentadecane as internal standard was analyzed directly by a gas chromatography flame ionization detector (GC-FID) (Agilent, United States). FAMEs were quantified by using a FAME mixture standard (Sigma-Aldrich, United States) with C17:0 as the internal standard. Lipid bodies were observed by using the Olympus BX53 fluorescence microscopy (Olympus, Japan). The algal cells were diluted to a density of 1 × 10^7^ cells mL^–1^ and then treated with 10% DMSO and stained for 10 min with 50 μM BODIPY 493/503 (Molecular Probes, Invitrogen Corporation, United States). Images were acquired by using the software cellSens DP6000 (Olympus, Japan). The BODIPY 493/503 fluorescence was detected using a 525/50 band-pass filter and the micrograph was taken using 488 nm excitation wavelength.

### Chlorophyll *a* Fluorometry Analysis

Photosynthetic activities were measured by using a pulse amplitude modulated (PAM) fluorometer (Dual-PAM 100, Walz, Effeltrich, Germany). Algal cells were dark-adapted for 15 min to measure the minimum fluorescence (F_0_). For the measurement of maximal fluorescence (Fm), a saturated pulse light (10,000 mmol photons m^–2^ s^–1^, lasting for 0.8 s) was applied to fully close the PSII reaction centers (Genty et al., 1989). Saturating flashes at intervals of 35 s were applied under the different actinic light intensities (0–2004 mmolm^–2^ s^–1^). The maximum fluorescence in the light (Fm’) and steady-state value of fluorescence (F) were recorded with saturation pulse analysis. The maximum photochemical efficiency of PSII (Fv/Fm) was calculated as (Fm-F_0_)/Fm. The quantum yield of PSII [Y(II)] and non-photochemical quenching (NPQ) were calculated as follows: Y(II) = (Fm’-F_0_)/Fm’, NPQ = (Fm-Fm’)/Fm’ (Maxwell and Johnson, 2000).

### RNA Extraction, Library Construction and Sequencing

Algal cells were collected at 6, 12, and 24 hours (h) of cultivation for the heterotrophically grown *S. acuminatus* cultivated under photoautotrophic lipid-inducing conditions (HL and NL) and heterotrophic conditions. For heterotrophic cultivation, 2 g L^–1^ of glucose was added into the N-limited BG-11 medium and the cells were grown under darkness. Mixing and aeration were provided by bubbling sterilized air at a flow rate of 0.2 vvm. For each time point, 3 biological replicates were prepared. Total RNA was extracted using TransZol Plant RNA Kit (TransGen, Beijing, China). For mRNA-Seq, the poly(A)-containing mRNA molecules were purified using NEBNext Poly(A) mRNA Magnetic Isolation Module (New England Biolabs). Directional transcriptome libraries were prepared using NEBNext Ultra Directional RNA Library Prep Kit for Illumina (New England Biolabs). Following the manufacturer’s instructions, RNA dissolved in the first strand synthesis reaction buffer and random primer mix were fragmented into 250- to 400-bp fragments by incubating the sample at 94°C for 10 min. The purified, fragmented and primed mRNA was converted into double-stranded cDNA. Then, adaptor ligation, purification of ligation reaction, PCR enrichment and purification of the PCR reaction were conducted, the final library was sequenced for 2 × 150-bp runs (paired-end) using Illumina Hiseq 2500 platform (Novogene Bioinformatics Technology Co., Ltd., Beijing, China).

### Transcriptome Assembly and Functional Annotation

To get high-quality clean reads, the raw data containing adaptor sequences, reads with low-quality sequences, and unknown nucleotides were filtered by using Trimmomatic (version 0.35). Transcriptome *de novo* assembly and quality assessment were performed firstly using Trinity (Grabherr et al., 2011), and the longest assembled transcript of given gene was taken as a unigene. Then the unigenes were used for CDS (Coding Sequence) prediction and functional annotation in the databases including: NR (NCBI non-redundant protein sequences), COG (Clusters of Orthologous Groups of proteins), GO (Gene Ontology) and KEGG (Kyoto Encyclopedia of Genes and Genomes) (Chen et al., 2017). Finally, the gene expression quantitation was estimated by RSEM (Li and Dewey, 2011) and each unigene was then calculated and normalized to the number of Fragments Per Kilobase Million (FPKM). Based on the expression, the differentially expressed genes (DEGs) were analyzed using the DEGseq R package (Huang et al., 2019). The significant DEGs were identified by two calculated parameters, false discovery rate (FDR) and Log_2_ fold change (Log_2_FC). If FDR was less than 0.05 and the absolute value of Log_2_FC was not less than 1, the gene was considered as significantly expressed one (Li X. et al., 2018).

### Statistical Analyses

All data were obtained by using at least three biological samples to ensure the reproducibility of the results. Experimental results were expressed as mean ± standard deviation. The data were analyzed by using one-way ANOVA in SPSS (version 19.0). Statistically significant difference was considered at *p* < 0.05.

## Results and Discussion

### Growth and FAMEs Contents of the HC and PC of *S. acuminatus* Subjected to the HL and NL Conditions

**FIGURE 1:**
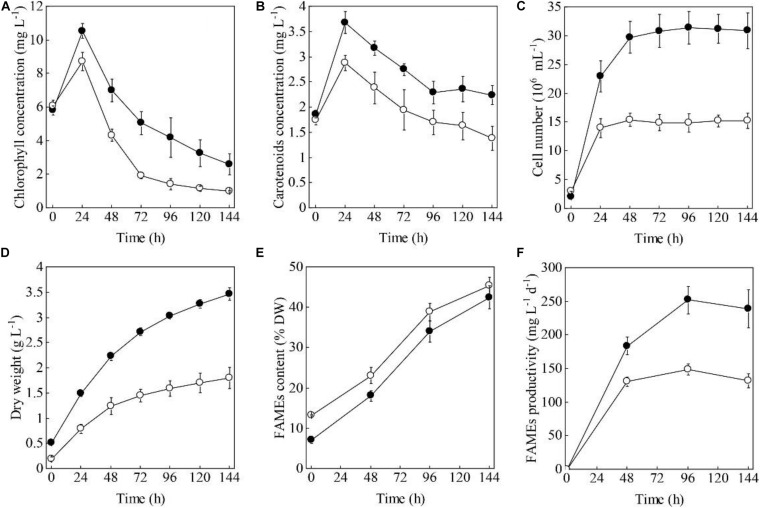
Time evolution of the volumetric chlorophyll concentrations **(A)**, carotenoid concentrations **(B)**, cell numbers **(C)**, dry weights **(D)**, FAMEs contents **(E)**, and FAMEs productivities **(F)** of the heterotrophically (filled cycle)- and photoautotrophically (empty cycle)-grown *S. acuminatus* cells subjected to the high-light and N-limited conditions. The values represent mean ± S.D. (*n* = 3).

When the HC and PC of *S. acuminatus* were subjected to the HL and NL conditions at the same initial chlorophyll concentration (ca. 6 mg L^–1^), the volumetric chlorophyll concentration of the HC culture increased more drastically than that of the PC culture within 24 h (Figure 1A). After 24 h, when the nitrate was completely consumed, the chlorophyll concentrations of both the cultures decreased. The chlorophyll concentration of the PC culture decreased more rapidly than that of the HC culture from 24 through 72 h under stresses (Figure 1A). Similarly, the volumetric carotenoid concentration of the HC culture doubled within 24 h and was much higher than that of the PC culture. After 24 h, the carotenoid concentrations of both the cultures decreased (Figure 1B).

When the two types of cells were inoculated at the same chlorophylls and carotenoids concentrations, the initial cell concentration was 2.1 × 10^6^ and 3.1 × 10^6^ cells mL^–1^, respectively, for the HC and PC culture (Figure 1C). The cell number of the HC culture reached 3.15 × 10^7^ cell mL^–1^ after 48 h under HL and NL stresses, which was about twice as many as that of the PC culture (1.49 × 10^7^ cell mL^–1^, *p* < 0.05, Figure 1C). The initial biomass concentration of the HC was twice as high as that of the PC (Figure 1D). Under the stress conditions, biomass yield of the HC culture was consistently higher than that of the PC culture during the 144 h under stresses (*p* < 0.05, Figure 1D). These results taken together indicated the HC can adapt to the HL and NL conditions in a more effective manner as compared to the PC.

To compare the capabilities in producing lipids of the two types of cells, the FAMEs contents and fatty acid profiles under HL and NL conditions were analyzed. As shown in Figure 1E, the initial FAMEs content of the HC was only 7.2% of DW, which was significantly lower than that of the PC (13.3% of DW) (*p* < 0.05). However, the FAMEs content of the HC increased sharply and reached 42.56% of DW by the end of cultivation, which was only a little lower than that of the PC (45.37% of DW). As a function of the biomass yield and the contents of FAMEs, the maximum FAMEs productivity of HC was significantly higher than that of PC under HL and NL conditions (*p* < 0.05, Figure 1F). Considering the total lipids of microalgae can be directly converted to the fatty acid methyl/ethyl esters for biofuels production (Nascimento et al., 2013; Sajjadi et al., 2018), the fatty acid profiles were compared between HC and PC. As shown in Supplementary Table 1, though the composition of PUFA was significantly higher in HC than that in PC, which was not suitable for biofuels manufacturing (Talebi et al., 2013), no significant difference in terms of the fatty acids profile was observed between them when HC and PC were subjected to HL and NL stresses over 144 h, indicating the coupled heterotrophy-and-photoautotrophy cultivation mode is an ideal technical route for biofuels production.

**FIGURE 2:**
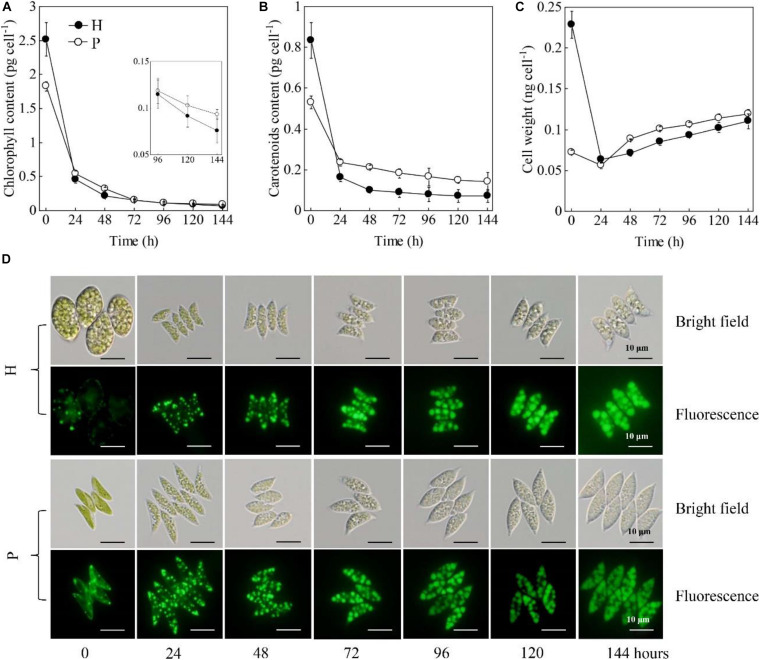
Changes in chlorophyll contents **(A)**, carotenoids contents **(B)**, cell weights **(C)**, cell morphologies and lipid bodies **(D)** of the heterotrophically (H)- and photoautotrophically (P)-grown *S. acuminatus* cells subjected to the high-light and N-limited conditions. Intracellular lipid bodies were stained using BODIPY (green). Scale bar = 10 μm.

The cellular contents of chlorophylls and carotenoids were significantly higher in the HC than that in the PC (*p* < 0.05, Figures 2A,B). The weight of the individual HC was 2 times higher than that of the PC (Figure 2C). These results were consistent with the microscopic observation that showed the size of HC (length 20.1 ± 3.0 μm, width 14.3 ± 2.5 μm) was significantly larger than that of PC (length 9.4 ± 2.1 μm, width 3.1 ± 0.4 μm) (Figure 2D). When subjected to the HL and NL conditions, the cellular contents of chlorophylls and carotenoids both decreased immediately within the 24 h under the stresses (Figures 2A,B). After 96 h, the chlorophyll content per cell and carotenoids content per cell of the HC were much lower than those of the PC, respectively (*p* < 0.05, Figures 2A,B). Upon the onset of the stresses, the weight per HC decreased from 0.23 to 0.06 ng cell^–1^ during the 24 h, while that of the PC slightly decreased (Figure 2C). After that, the weight of the two types of cells continuously increased and no significant difference was observed between them (*p* > 0.05, Figure 2C). Decreases in the weight per HC were consistent with the changes in cell morphology as shown in Figure 2D. Besides, the number and size of lipid bodies in the two types of cells were almost the same (Figure 2D). In addition, the calculated contents of chlorophylls and carotenoids based on cell dry weight were shown in Supplementary Table 2. The results showed that the chlorophyll content of HC and PC decreased to a comparable level under HL and NL stress conditions, though it was originally two times higher in PC than that in HC. By contrast, the carotenoid content per cell dry weight of PC was 98.6% higher than that of HC. When subjected to the stresses, the carotenoid content of PC decreased by 42.3% within 24 h, while that of HC decreased by 29.6%. Over 144 h under HL and NL stresses, the carotenoid contents of PC were constantly higher than that of HC.

Those results taken together indicated HC had the more or less same ability in accumulating lipids with the PC but much higher growth rate under HL and NL conditions. Different from many previous studies, which investigated the microalgal biomass and lipid yields during the trophic transition process (i.e., from heterotrophy to photoautotrophy) (Han et al., 2012; Wu et al., 2019), this study uncovered the changes in the morphology and biochemical compositions at the single cell level. Firstly, it was observed that both the HC and PC of *S. acuminatus* started to divide vigorously upon being subjected to the high light conditions, and the HC divided to more extent than PC did. After 24 h, when the nitrate in the growth media was completely consumed up, the division of PC immediately stopped but the division of HC continued until 48 h, albeit at a slightly lower rate than that of the first 24 h under HL stress. Though the original weight of HC was much heavier than that of PC, it decreased to a comparable level with that of PC over 144 h under HL and NL stresses. In the previous study, the enhanced growth rate of HC under HL and NL conditions was attributable to its remarkably reduced chlorophyll content (Jin et al., 2020). It was suggested that HC may possess truncated light-harvesting antennae, which can permit higher light penetration in high-cell-density-culture, less likelihood photoinhibition, and reduced energy loss as heat (Melis, 2009; Cazzaniga et al., 2014). However, it was found in this study that the chlorophyll contents of the PC and HC were reduced to a similar level under stresses (Figure 2 and Supplementary Table 1). By contrast, it was observed that the cellular content of carotenoids of PC was higher than that of HC. Enhancement of the carotenoid biosynthesis is a strategy adopted by microalgae to cope with the excess light (Li L. et al., 2018). Thus, we assumed the relatively lower cell concentration of the PC culture led to the exposure of the single cells to excess illumination and therefore the retarded cell growth of PC under HL and NL stresses. Thus, understanding the physiological and biochemical changes that occurred in HC during the first 48 h under stresses, which caused rapid cell division, is crucial for elucidating the mechanisms underlying the enhanced growth rate of HC. On the other hand, no significant difference was observed in the lipid contents between PC and HC. Previous studies on *Chlorella* spp. suggested that the lipid contents of the HC subjected to HL stress was significantly lower than that of PC under the same conditions (Han et al., 2012). Thus, the distinct capabilities in accumulating lipids between HC and PC subjected to the same stresses may vary among microalgal species.

### Photosynthetic Efficiencies of the HC and PC Subjected to HL and NL Conditions

**FIGURE 3:**
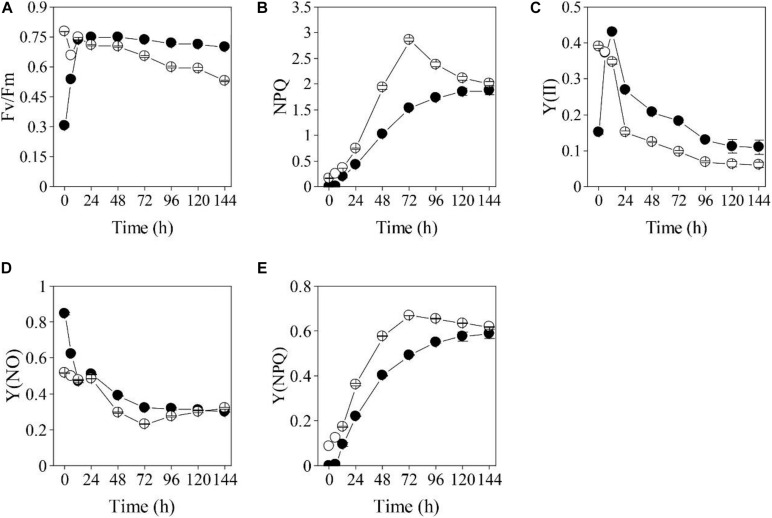
Changes in chlorophyll fluorometry parameters [**(A):** Fv/Fm, **(B):** NPQ, **(C):** Y(II), **(D):** Y(NO), **(E):** Y(NPQ)] for the heterotrophically (filled cycle)- and photoautotrophically (empty cycle)-grown *S. acuminatus* cells subjected to the high-light and N-limited conditions. The values represent mean ± S.D. (*n* = 3).

The impacts of the HL and NL stresses on the photosynthetic efficiencies of the two types of *S. acuminatus* cells were investigated by using the chlorophyll *a* fluorometry (Figure 3). The initial Fv/Fm of the PC was 0.78 (Figure 3A). However, the Fv/Fm of the PC decreased sharply after 24 h of cultivation under HL and NL conditions. On the contrary, although the initial Fv/Fm of the HC was only 0.31, which suggested that the photosynthetic complexes were not well developed or impaired in HC, it increased sharply during the first 12 h of cultivation and reached the maximum value of 0.75 at 24 h. Afterward, Fv/Fm of the HC showed a slight decrease during cultivation and it was much higher than that of the PC under the same conditions (*p* < 0.05, Figure 3A). Similar results were obtained in effective PSII quantum yield [Y(II)] (Figure 3C).

On the other hand, non-photochemical quenching (NPQ) of the PC subjected to HL and NL was greatly induced, and was significantly higher than that of the HC from 24 through 96 h of stresses (*p* < 0.05, Figure 3B), which was consistent with the results of the yield for dissipation by downregulation [Y(NPQ)] (Figure 3E). Although enhanced NPQ is a useful strategy for microalgal cells to cope with the excess light (Peers et al., 2009; Bailleul et al., 2010), more light energy dissipates as heat may cause less quantum yield. Thus, much lower NPQ of the HC than that of PC may lead to higher light energy utilization by the former one, which results in its higher growth rate and biomass yield under stresses. The yield of other non-photochemical losses [Y(NO)] of the cells in the two cultures were almost the same after 24 h of cultivation (Figure 3D).

**FIGURE 4:**
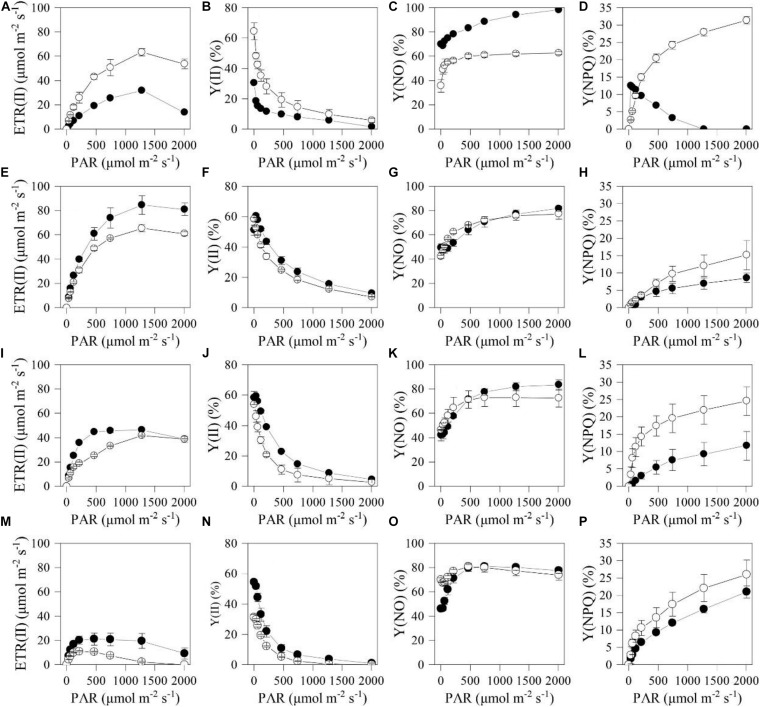
Light response curves of the chlorophyll fluorometry parameters for the heterotrophically (filled cycle)- and photoautotrophically (empty cycle)-grown *S. acuminatus* cells subjected to the high-light and N-limited conditions. Chlorophyll fluorescence were obtained with a series of photosynthetically active radiances (0, 34, 65, 122, 218, 467, 746, 1279, 2004 μmol photons m^–2^ s^–1^) to calculate ETR(II), Y(II), Y(NO) and Y(NPQ). **(A–D)**, **(E–H)**, **(I–L)**, and **(M–P)** were measured in 0, 12, 24, and 144 h of cultivation, respectively. The values represent mean ± S.D. (*n* = 3).

To better understand the differences in photosynthetic physiology between the two types of cells, light-response curves were determined for them. The PC exhibited higher electron transport rate (ETR) and Y(II) than HC under the actinic light in the range of 34–2004 μmol photons m^–2^ s^–1^ (Figures 4A,B). However, those two parameters of the HC were much higher than that of the PC under the actinic light in the range of 34–1279 μmol photons m^–2^ s^–1^ after 12 h (Figures 4E,F,I,J,M,N). Moreover, Y(NPQ) of the PC subjected to HL and NL was higher than that of HC under the same stress conditions (Figures 4D,H,L,P), while Y(NO) of the two types of cell subjected to the stresses for 12 h had little disparity under the actinic light in the range of 65–2004 μmol photons m^–2^ s^–1^ (Figures 4C,G,K,O).

The heterotrophically grown algal cells are usually featured by the underdeveloped or impaired photosystems, indicated by the relatively low Fv/Fm value (Zhang et al., 2016; Roth et al., 2019). The Fv/Fm of *Haematococcus pluvialis* cells grown under heterotrophic conditions is found to be as low as 0.6. When *Chlorella protothecoides* was grown under heterotrophic conditions, many photosynthetic proteins were found to be degraded, reflecting changes in photosynthetic efficiencies (Gao et al., 2014). Different from photosynthetic cells of many microalgae, whose photosynthetic efficiencies dramatically declined when they are subjected to the HL stresses (Parkhill et al., 2010; Xia et al., 2014; He et al., 2015), the Fv/Fm and Y(II) and of HC immediately recovered during the trophic transition of *S. acuminatus* (Figure 3). Underlying mechanism for the rapid regeneration and reactivation of photosynthesis may be involved in removal of glucose that can suppress photosynthesis in HC (Roth et al., 2019). In addition, both the light and nitrate were found to be capable of inducing reconstruction of photosynthetic machine of heterotrophic algal cells (Zhang et al., 2016). The results of chlorophyll a fluorometry underlined the significance of changes during the first 24 h, which may confer the HC of *S. acuminatus* capabilities in adapting to the HL and NL conditions. The results also suggested that more light energy can be converted into photochemical energy and less light energy dissipated as heat in the HC than those in the PC, which could lead to the enhanced biomass production of HC under the HL and NL conditions.

### Overview of the RNA-Seq Data

To dissect the mechanisms underlying the enhanced biomass and lipid production of heterotrophically grown *S. acuminatus* cells under stress conditions, transcriptomics analysis was performed for the HC subjected to photoautotrophic lipid-induing conditions (HL and NL) and the cells were kept under the heterotrophic conditions were used as control. After assembling the *de novo* sequenced transcripts and eliminating redundancy, 15,899 unigenes with an N50 length of 1,193 bp and GC content of 55.6% were obtained (Supplementary Table 3). To evaluate the accuracy and completeness of assembled unigenes, the transcripts were compared with single-copy gene sets of several large evolutionary branches in OrthoDB database^1^ by BUSCO 3.0 (Simao et al., 2015), based on conservation of single-copy benchmarking universal single-copy orthologs (BUSCOs). The results showed a high degree of completeness with a BUSCO score of 76.5%, of which 1,659 genes were complete, 525 were complete duplicated, 230 were fragmented, and 279 were missing BUSCO orthologs out of the 2,168 groups searched (Supplementary Table 3). These results indicated high-quality transcriptomes were obtained in this study and can be further used for annotation and analysis.

**FIGURE 5:**
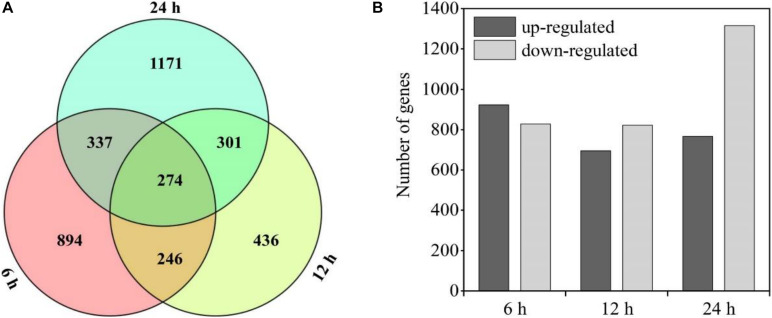
Statistical chart of heterotrophically grown *S. acuminatus* cells subjected to the high-light and N-limited conditions. Venn diagram representation of shared/unique genes **(A)** and number of differently expressed genes (DEGs) **(B)** compared to heterotrophically grown cells at 6, 12, and 24 h of cultivation, respectively.

There were 274 shared genes identified in both the treated (HC subjected to the HL and NL conditions) and control groups at three time points (Figure 5A). Besides, there were 5,351 DEGs in the heterotrophically grown *S. acuminatus* cells subjected to HL and NL as compared to the control. Among them, 923, 695, and 767 up-regulated and 828, 822, and 1,316 down-regulated DEGs were identified at 6, 12, and 24 h under the HL and NL conditions (Figure 5B). However, much more DEGs were observed when heterotrophically grown *C. pyrenoidosa* and *C. zofinginesis* were transferred to photoautotrophic conditions (Fan et al., 2015; Roth et al., 2019).

The unigenes were further classified by GO enrichment analysis and KEGG enrichment pathway analysis (Supplementary Figures 1–3). Notably, in the early stage of trophic transition (6 h), the transcript levels of the genes related to membrane and membrane part, which involved in chloroplast biosynthesis, were fluctuated remarkably (Supplementary Table 4). These findings suggested a large number of biological processes were reprogrammed in the HC of *S. acuminatus* during the trophic transition, which was also observed during the trophic transition processes in *C. pyrenoidosa* and *C. zofinginesis* (Fan et al., 2015; Roth et al., 2019).

### Changes in the Key Biological Processes at Transcriptional Level During the Trophic Transition

Previous studies have revealed that a large number of pathways including photosynthesis, carbon fixation, central carbon metabolism, oxidative phosphorylation, lipid biosynthesis, and other metabolism pathways were regulated at the transcriptional level during the trophic transition process in many microalgae, which enabled microalgal cells coping with the changing environmental conditions (Fan et al., 2015; Roth et al., 2019). Photosynthesis produce the main energy sources (i.e., ATP and NADPH) for the growth and metabolism of algal cells (Huang et al., 2019). When dark-grown algal cells were subjected to HL condition, photosynthesis may be recovered immediately to provide energy for sustaining the algal cell growth. Four multi-subunit membrane-protein complexes in the thylakoid membranes including two photosystems (PSI and PSII), cytochrome b6f and ATPase were the basic elements for photoreaction (Nelson and Ben-Shem, 2004). The results showed that 37 genes coding for the above four photosynthetic apparatus were differently expressed at 6 h (Table 1). D1 protein turnover is an important defense strategy for plants and algae to cope with HL stresses (Wu et al., 2011; Li L. et al., 2018). The transcriptome data showed that although the D1 protein encoding gene psbA (c11222_c0_g1) was downregulated by 1.38-fold at 6 h, it was upregulated by 95 and 38% at 12 and 24 h, respectively (Table 1). This result suggested that D1 protein of the heterotrophically grown cells subjected to HL and NL can be repaired so as to enhance the function of PSII, which was consistent with the increase of Fv/Fm and Y(II) of algal cells during the same period of cultivation time (Figures 3A,C). Non-photochemical quenching of excess excitation energy is another photoprotective strategy in photosynthetic organisms (Correa-Galvis et al., 2016). Several studies have already confirmed that high quenching capacity depending on PsbS (Gerotto et al., 2015; Ware et al., 2015; Correa-Galvis et al., 2016). The gene (c19487_c0_g1) encoding PsbS was downregulated by 31% and 1.19 times at 6 and 12 h, respectively (Table 1). However, it was upregulated by 63% at 24 h. This result indicated NPQ was enhanced with extended culturing time under HL and NL stresses, which was consistent with changes in the NPQ during the same period of time (Figures 3B,E). LHCII is the primary site of photoprotection and its recovery is also useful for algal cells to eliminate photodamage caused by HL (Ruban et al., 2012). D1 protein turnover and PsbS upregulation indicated that some key proteins in LHCII were repaired under stresses, and it may provide protection for the core of PSII to some extent. Thus, downregulation of the expression of the genes involved in photosynthesis indicated that the low concentration of glucose (i.e., 0–5 g L^–1^) adopted in this study may stimulate expression of many photosynthetic genes in the *S. acuminatus* cells grown under the heterotrophic conditions.

**TABLE 1:** Comparative transcriptomic analysis of photosynthesis for the heterotrophically grown *S. acuminatus* cells subjected to the high light and N-limited conditions.

Category	Gene_ID	KO_ID	Enzyme	Gene_name	6 h		12 h		24 h	
					Log_2_FC	FDR	Log_2_FC	FDR	Log_2_FC	FDR
PSI	c11348_c0_g1	K02689	Photosystem I P700 chlorophyll a apoprotein A1	PsaA	–1.763	3.78E-16	–1.107	2.32E-04	–0.373	2.71E-01
	c2811_c0_g1	K02692	Photosystem I subunit II	PsaD	0.138	2.43E-03	–0.513	1.58E-14	–2.950	7.93E-272
	c16988_c0_g1	K08905	Photosystem I subunit V	PsaG	0.208	4.28E-04	0.094	2.74E-01	–2.539	4.22E-139
	c13333_c0_g1	K02696	Photosystem I subunit VIII	PsaI	0.217	1.94E-07	–0.442	2.46E-09	–3.678	4.01E-209
	c7310_c0_g1	K02699	Photosystem I subunit XI	PsaL	0.194	3.85E-06	–0.177	3.48E-02	–3.780	1.22E-272
	c6347_c0_g1	K02701	Photosystem I subunit PsaN	PsaN	0.947	2.67E-80	0.513	5.83E-12	–4.628	7.28E-306
	c15041_c0_g1	K02639	Ferredoxin	PetF	–0.622	7.13E-18	–1.825	6.23E-73	–2.927	3.64E-88
	c19224_c0_g3	K02638	Plastocyanin	PetE	–0.536	8.02E-51	–0.508	2.51E-11	–2.378	2.66E-131
	c16349_c0_g1	K02641	Ferredoxin-NADP + reductase	PetH	0.296	8.13E-13	0.027	7.58E-01	–1.821	9.11E-131
PSII	c11222_c0_g1	K02703	Photosystem II P680 reaction center D1 protein	PsbA	–1.383	2.23E-47	0.948	6.97E-07	0.375	2.69E-02
	c14164_c0_g1	K02704	Photosystem II CP47 chlorophyll apoprotein	PsbB	–2.446	1.49E-20	–2.224	4.76E-09	NA	NA
	c15153_c0_g1	K02705	Photosystem II CP43 chlorophyll apoprotein	PsbC	–1.006	1.30E-09	0.659	2.53E-03	–0.742	3.99E-04
	c2432_c0_g1	K02706	Photosystem II P680 reaction center D2 protein	PsbD	–2.088	5.01E-18	NA	NA	NA	NA
	c17184_c0_g1	K02716	Photosystem II oxygen-evolving enhancer protein 1	PsbO	0.471	2.62E-37	–0.236	5.32E-04	–3.520	0.00E + 00
	c17823_c0_g1	K02717	Photosystem II oxygen-evolving enhancer protein 2	PsbP	0.196	2.66E-07	–0.052	4.41E-01	–3.598	3.68E-291
	c16649_c0_g1	K08901	Photosystem II oxygen-evolving enhancer protein 3	PsbQ	0.587	6.94E-43	0.028	7.71E-01	–3.889	7.78E-247
	c18753_c1_g2	K03541	Photosystem II 10 kDa protein	PsbR	0.028	5.36E-01	–0.467	2.38E-09	–2.686	1.44E-233
	c19487_c0_g1	K03542	Photosystem II 22 kDa protein	PsbS	–0.310	9.70E-02	–1.194	6.65E-14	0.626	4.83E-03
	c18171_c0_g1	K02723	Photosystem II PsbY protein	PsbY	1.049	8.29E-108	–0.358	3.63E-06	–3.184	6.70E-154
	c15459_c0_g1	K08902	Photosystem II Psb27 protein	Psb27	0.436	2.85E-11	–0.365	6.61E-06	–2.292	9.67E-76
	c19359_c6_g1	K08903	Photosystem II 13 kDa protein	Psb28	–0.959	2.20E-05	–1.892	1.42E-20	–5.144	7.84E-56
LHC	c19400_c0_g1	K08907	Light-harvesting complex I chlorophyll a/b binding protein 1	LHCA1	–2.329	0.00E + 00	–1.931	1.70E-184	–2.795	0.00E + 00
	c13244_c1_g1	K08908	Light-harvesting complex I chlorophyll a/b binding protein 2	LHCA2	–0.024	5.98E-01	0.287	1.86E-04	–3.787	0.00E + 00
	c14924_c0_g1	K08909	Light-harvesting complex I chlorophyll a/b binding protein 3	LHCA3	–0.046	2.99E-01	0.156	2.54E-02	–4.177	0.00E + 00
	c12657_c0_g1	K08910	Light-harvesting complex I chlorophyll a/b binding protein 4	LHCA4	0.177	6.58E-06	–0.044	5.73E-01	–4.682	0.00E + 00
	c13616_c0_g1	K08911	Light-harvesting complex I chlorophyll a/b binding protein 5	LHCA5	0.211	3.72E-09	0.064	4.39E-01	–3.896	0.00E + 00
	c19523_c4_g1	K08912	Light-harvesting complex II chlorophyll a/b binding protein 1	LHCB1	0.368	9.03E-20	0.330	1.94E-06	–3.863	0.00E + 00
	c19022_c2_g6	K08915	Light-harvesting complex II chlorophyll a/b binding protein 4	LHCB4	0.354	1.75E-19	–0.194	1.25E-02	–3.417	3.08E-209
	c12740_c0_g1	K08916	Light-harvesting complex II chlorophyll a/b binding protein 5	LHCB5	–0.285	1.08E-10	–0.009	9.30E-01	–4.435	0.00E + 00
ATPase	c12514_c0_g1	K02109	F-type H + -transporting ATPase subunit b	ATPF0B	–0.216	1.69E-07	–0.523	9.20E-16	–3.084	0.00E + 00
	c19297_c1_g1	K02113	F-type H + -transporting ATPase subunit delta	ATPF1D	–0.295	1.91E-12	–0.329	4.95E-07	–3.131	0.00E + 00
	c19650_c0_g1	K02115	F-type H + -transporting ATPase subunit gamma	ATPF1G	0.111	1.51E-02	–0.493	2.05E-12	–2.715	4.64E-186
b6f	c19562_c6_g4	K08906	Cytochrome c6	PetJ	–0.689	1.45E-07	–1.521	2.04E-27	–3.483	8.69E-31
	c16278_c0_g2	K02636	Cytochrome b6-f complex iron-sulfur subunit	PetC	0.452	8.79E-27	–0.220	2.59E-03	–2.793	2.66E-231

The genes involved in carotenoid biosynthesis were also surveyed. As shown in Table 2, the gene encoding phytoene synthase (PSY), which catalyzes the conversion of geranylgeranyl diphosphate to phytoene, was downregulated significantly at all three time points (Table 2). The major carotenoids of *S. acuminatus* are lutein, zeaxanthin, violaxanthin, and neoxanthin (Zhang et al., 2019). The expressions of corresponding genes were all downregulated, including beta-ring hydroxylase (CYP97A3) and carotenoid epsilon hydroxylase (CYP97C1), beta-carotene 3-hydroxylase (CHYB) and violaxanthin de-epoxidase (VDE) (Table 2). These results indicated the carotenoid biosynthesis of *S. acuminatus* was downregulated at the gene expression level during the trophic transition process, which was consistent with the carotenoid quantification results shown in Figure 2B. Because lutein, zeaxanthin, violaxanthin and neoxanthin are four main carotenoids of the xanthophyll cycle (Janik et al., 2016), the down-regulation of corresponding genes may also decrease xanthophyll cycle and finally generate lower NPQ, which was confirmed by the results achieved in Figure 3B.

**TABLE 2:** Comparative transcriptomic analysis of carotenoid biosynthesis for the heterotrophically -grown *S. acuminatus* cells subjected to the high light and N-limited conditions.

Gene_ID	KO_ID	Enzyme	Gene_name	6 h		12 h		24 h	
				Log_2_FC	FDR	Log_2_FC	FDR	Log_2_FC	FDR
c4751_c0_g1	K00514	Zeta-carotene desaturase	ZDS	–1.663	1.72E-59	–1.134	2.13E-32	–1.669	1.18E-42
c5887_c0_g1	K02291	Phytoene synthase	PSY	–1.985	3.22E-30	–3.587	4.94E-104	–4.356	2.92E-75
c6666_c0_g1	K09839	Violaxanthin de-epoxidase	VDE	0.239	5.88E-02	0.089	6.17E-01	–1.587	1.70E-06
c6677_c0_g1	K09838	Zeaxanthin epoxidase	ZEP	0.247	2.96E-04	–0.606	6.36E-13	0.021	8.49E-01
c7062_c5_g1	K06444	Lycopene epsilon-cyclase	LCYE	–1.518	4.14E-15	–2.820	3.13E-40	–3.642	3.45E-14
c7246_c6_g1	K06443	Lycopene beta-cyclase	LCYB	–1.736	4.89E-09	–0.407	4.41E-02	–1.213	1.74E-04
c7438_c7_g1	K09836	Beta-carotene/zeaxanthin 4-ketolase	BKT	–1.105	2.42E-22	–1.203	4.71E-16	–0.049	8.33E-01
c7533_c5_g3	K09843	(+)-abscisic acid 8′-hydroxylase	CYP707A	–0.075	7.97E-01	1.086	1.96E-04	2.393	3.11E-10
c7684_c6_g6	K09843	(+)-abscisic acid 8′-hydroxylase	CYP707A	–0.989	5.48E-50	–0.738	2.81E-17	–0.239	6.21E-02
c7728_c5_g5	K15747	Beta-ring hydroxylase	CYP97A3	–1.097	1.36E-04	–1.876	4.64E-15	–3.293	2.94E-19
c7760_c4_g2	K09837	Carotenoid epsilon hydroxylase	CYP97C1	–2.136	3.69E-24	–2.080	4.15E-15	NA	NA
c7813_c9_g1	K15745	Phytoene desaturase (3,4-didehydrolycopene-forming)	PDS	–1.884	2.28E-12	–1.884	5.20E-07	NA	NA
c8132_c5_g1	K15746	Beta-carotene 3-hydroxylase	CHYB	0.287	3.37E-01	–2.342	1.29E-24	–3.858	2.42E-22
c8212_c6_g1	K15744	Zeta-carotene isomerase	ZISO	–2.038	1.65E-28	–0.906	4.31E-05	NA	NA
c8551_c2_g1	K02293	Phytoene desaturase	PDS	–1.271	7.44E-84	–0.757	1.42E-24	–2.526	2.40E-181

*Log_2_FC is Log2 Fold Change. Positive value means upregulated and negative value means downregulated, NA means without detected gene.*

Photosynthetic carbon fixation can provide energy and carbon skeletons for biosynthesis of macromolecules. Ribulose-1,5-bisphosphate carboxylase/oxygenase (RuBsiCO) catalyzes the addition of gaseous carbon dioxide to ribulose-1,5-bisphosphate (RuBP), generating two molecules of 3-phosphoglyceric acid, is the key enzyme involved in photosynthetic carbon fixation. The transcriptomics analysis revealed that two transcripts encoding RuBsiCO were differently expressed during trophic transition. One transcript (c8501_c0_g1) encoding the large subunit (rbcL) of RuBsiCO was downregulated by 1.95 and 1.65 times at 6 and 24 h, respectively. The transcript (c13567_c0_g1) encoding the small subunit (rbcS) of RuBsiCO was downregulated by 2.94 times at 24 h (Supplementary Table 5). Though downregulation of RuBsiCO under HL and NL may reduce the efficiency of Calvin cycle, the carbon assimilation in *S. acuminatus* cells CO_2_ fixation may be compensated by other processes. Despite that RuBisCO is the main primary CO_2_-fixing enzyme in algae and C3 plants, these organisms also possess a second enzyme, phosphoenolpyruvate carboxylase (PEPC) that can efficiently fix carbon as it catalyzes the reaction of CO_2_ and phosphoenolpyruvic acid to produce oxaloacetic acid (Chen et al., 2002; Durall and Lindblad, 2015). The results showed that the genes (c13707_c0_g1, c48143_c0_g1) encoding PEPC was up-regulated at 6 and 12 h (Supplementary Table 5). Moreover, another key enzyme belonging to C4 and Crassulacean Acid Metabolism (CAM) pathways for carbon fixation, pyruvate orthophosphate dikinase (PPDK), was up-regulated significantly at three time-points at the transcript level (Supplementary Table 5).

**FIGURE 6:**
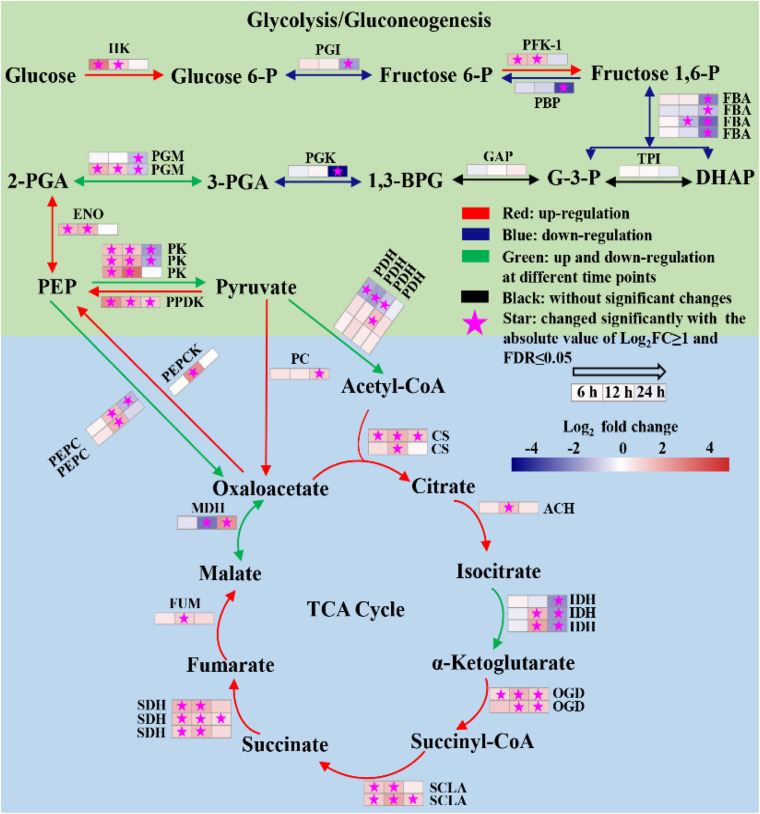
Reconstructed glycolysis/gluconeogenesis and TCA cycle of heterotrophically grown *S. acuminatus* cells subjected to the high-light and N-limited conditions. Genes upregulated were indicated in red. Genes downregulated were indicated in blue. Genes upregulated and downregulated at different time points were indicated in green. No significant changes were indicated in black. The full names of the corresponding genes are given in Supplementary Table 6.

The glycolysis/gluconeogenesis pathway, pentose phosphate pathway (PPP) and the tricarboxylic acid (TCA) cycle were the central metabolic pathways of carbon metabolism in microalgae (Fan et al., 2015; Fan et al., 2016; Lv et al., 2019). In this study, the changes in the transcripts belonging to the central carbon metabolism are listed in Supplementary Table 6. Based on the data, the transcriptional regulation on glycolysis/gluconeogenesis and TCA cycle was constructed in Figure 6. The results showed that most genes involved in glycolysis/gluconeogenesis were remarkably upregulated at 6 and 12 h, including those encoding hexokinase (HK), 6-phosphofructokinase-1 (PFK-1), phosphoglucomutase (PGM), enolase (ENO), pyruvate kinase (PK) and pyruvate orthophosphate dikinase (PPDK). Besides, acetyl-CoA synthetase (ACS), which catalyzes production acetyl-CoA from acetate, was also significantly upregulated at 6 and 12 h (Supplementary Table 6). As acetyl-CoA is a precursor in the glyoxylate cycle, TCA cycle, and fatty acid biosynthesis, the upregulation of ACS may enhance the carbon flux in these pathways during the trophic transition process.

Tricarboxylic acid cycle produces NADH and FADH2 in aerobic organisms for carbon metabolism (Lv et al., 2019). As shown in Figure 6, most genes encoding key enzymes in TCA cycle were significantly upregulated at all three time points, including citrate synthase (CS), aconitate hydratase (ACH), 2-oxoglutarate dehydrogenase (OGDH), succinate dehydrogenase (SDHA), succinate dehydrogenase (SDH) and fumarate hydratase (FUM). It indicated that the TCA cycle was significantly enhanced of the heterotrophically grown *S. acuminatus* cells subjected to HL and NL. Besides, most genes participated in electron transfer chains and oxidative phosphorylation were significantly upregulated at 6 and 12 h (Supplementary Table 7). Thus, NADH and FADH2 produced by TCA cycle could be used effectively through electron transfer chains and oxidative phosphorylation to form ATP. Therefore, enhanced TCA cycle and oxidative phosphorylation could be beneficial to produce reducing power and ATP for maintaining rapid growth of *S. acuminatus* cells during the trophic transition.

The elevated PPP activity was often induced by photooxidative stress and accompanied by the production of reactive oxygen species (ROS) (Zheng et al., 2017). Most enzymes in PPP were downregulated at the gene expression level at all three time points (Supplementary Table 6). In addition, it was observed that a number of antioxidant enzymes (i.e., glutathione peroxidase, catalase, iron-superoxide dismutase) encoding genes were downregulated during the trophic transition (Supplementary Table 8). Co-downregulation of the PPP and antioxidant enzymes suggested that no severe photodamage occurred in the algal cells during the trophic transition.

**FIGURE 7:**
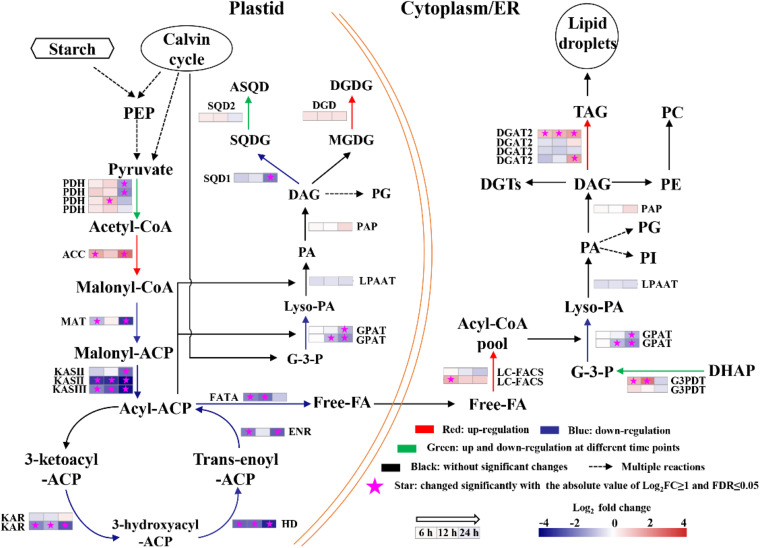
Changes in transcript abundance of genes involved in fatty acids biosynthesis of heterotrophically grown *S. acuminatus* cells subjected to the high-light and N-limited conditions. Genes upregulated were indicated in red. Genes downregulated were indicated in blue. Genes upregulated and downregulated at different time points were indicated in green. No significant changes were indicated in black. The full names of the corresponding genes are given in Supplementary Table 9.

Fatty acid biosynthesis and glycerolipid biosynthesis pathways are responsible for converting the photosynthetically fixed carbon to triacylglycerols (TAGs) and membrane lipids. The transcriptomics data showed that although the gene encoding the acetyl-CoA carboxylase/biotin carboxylase (ACCase), which is the rate limiting enzyme of the fatty acid *de novo* biosynthesis pathway, was up-regulated at all three time points, whereas the other genes involved in fatty acid biosynthesis were significantly down-regulated (Figure 7). In addition, it was observed that the gene encoding digalactosyl diacylglycerol synthase (DGD), which catalyzes monogalactosyl diglyceride to digalactosyl diacylglycerol (DGDG), was up-regulated in the lipid biosynthesis pathway at 6, 12, and 24 h (Supplementary Table 9). DGDG is the main bilayer lipid of the thylakoid membranes of microalgae and plays an important role in maintaining the normal fluidity of thylakoid membrane (Sakurai et al., 2007). Therefore, the up-regulation of DGD may be beneficial to the reconstruction of photosynthetic membranes of *S. acuminatus* cells during the trophic transition. Besides, one copy of diacylglycerol acyltransferase 2 (DGAT2) was significantly upregulated at 6, 12, and 24 h (Figure 7). DGAT catalyzes the last step of triacylglycerol biosynthesis and its upregulation can enhance lipid production (Sharma and Chauhan, 2016). The identified DGAT encoding gene responsive to the trophic transition could be a target for genetic engineering to further increase the lipid production under the coupled heterotrophic and photoautotrophic cultivation mode. Such a limited number of DEGs were found to be involved in TAG assembly during the trophic transition process, reflecting unresponsive gene expression within the timeframe of this study. In addition, it is noteworthy that the expression patterns of most TAG assembly related genes are not correlated well with the TAG accumulation in microalgae, with the exception of a few copies of the DGAT genes (Blaby et al., 2013; Li et al., 2014). However, in *C. zofinginesis*, the expression of most genes responsible for TAG assembly, including those coding for glycerol-3-phosphate: acyl-CoA acyltransferase, lysophosphatidic acid: acyl-CoA acyltransferase, and phosphatidic acid phosphatase, are found to be congruent with TAG accumulation and degradation during the trophic transition process (Roth et al., 2019). Thus, responses of TAG biosynthesis during trophic transition remain to be an important area for future investigation. Moreover, TAG biosynthesis often accompanied by starch degradation in many microalgae (Wu et al., 2019). The transcriptomics data also showed that the genes encoding starch synthase were significantly downregulated at 24 h of cultivation (Supplementary Table 10), indicating the carbon fluxes were channeled to lipid synthesis. Besides, several genes involved in nitrogen metabolism were upregulated significantly at 24 h of cultivation, including nitrate/nitrite transporter, nitrate reductase [NAD(P)H], ferredoxin-nitrite reductase and glutamine synthetase (Supplementary Table 11), suggesting the nitrogen assimilation was enhanced in HC under stresses, which could provide precursors and energy for lipid biosynthesis.

Among the identified DEG, there were 8 very-long-chain fatty acid synthase encoding genes showing significant up-regulation at multiple time points from heterotrophy to photoautotrophy conditions (Table 3). Similar results were obtained from *C. zofinginesis*, of which 3 ketoacyl-CoA synthase encoding genes were significantly up-regulated when the algal cells were shifted from heterotrophy to photoautotrophy (Roth et al., 2019). Very-long-chain fatty acids are suggested to be the acyl groups of wax in many microalgae and land plants and to reduce the photodamage to cells under HL (Kondo et al., 2016; Rashidi and Trindade, 2018). However, it remains to be investigated whether *S. acuminatus* could synthesize wax under stress conditions in future studies.

**TABLE 3:** Changes in transcript expression of several genes related to very-long-chain fatty acids synthesis for the heterotrophically grown *S. acuminatus* cells subjected to the high-light and N-limited conditions.

Gene_ID	Enzyme	6 h		12 h		24 h	
		Log_2_FC	FDR	Log_2_FC	FDR	Log_2_FC	FDR
c19395_c0_g1	3-ketoacyl-CoA synthase 6	2.670	0.00E + 00	–0.085	1.61E-01	0.901	5.74E-32
c19313_c4_g2	3-ketoacyl-CoA synthase 9	3.041	1.50E-119	0.114	1.76E-01	0.627	1.72E-06
c19313_c4_g12	3-ketoacyl-CoA synthase 1	2.602	2.17E-72	–0.197	1.99E-02	0.760	1.42E-06
c10824_c0_g1	3-ketoacyl-CoA synthase 11	2.226	3.94E-56	1.111	1.71E-39	1.774	1.10E-121
c37974_c0_g1	3-ketoacyl-CoA synthase 11	2.193	7.05E-32	1.064	4.83E-18	2.193	7.05E-32
c34341_c0_g1	3-ketoacyl-CoA synthase 19	2.088	2.59E-36	1.184	1.01E-32	2.015	3.74E-114
c19313_c4_g5	3-ketoacyl-CoA synthase 19	1.142	8.09E-07	2.145	2.58E-34	5.245	0.00E + 00
c11396_c1_g1	3-ketoacyl-CoA synthase 19	1.690	3.23E-06	2.423	8.14E-25	5.566	1.16E-267

*Log_2_FC is Log2 Fold Change. Positive value means upregulated and negative value means downregulated.*

## Conclusion

In this study, we demonstrated that the heterotrophically grown *S. acuminatus* cells possessed the advantages over the photoautotrophically grown cells in terms of biomass and lipid production when subjected to HL and NL conditions. Under the stress conditions, the Fv/Fm and Y(II) of the heterotrophically grown cells were recovered to the maximum values after 24 h and were much higher than the photoautotrophically grown counterparts. Transcriptomic analysis revealed that heterotrophically grown cells fully expressed the photosystems encoding genes and the low concentration of glucose may stimulate the expression of a number of genes involved in photosynthesis. Moreover, regulation of a number of pathways involved in carbon metabolism was deduced to provide sufficient energy for sustaining vigorous growth of *S. acuminatus* cells under stresses. Enhanced lipid production may be attributable to the upregulation of ACCase and DGAT2 at the gene expression level. Our findings shed light on the mechanisms underlying the enhanced growth and lipid production in the algal cells during the trophic transition process.

## Data Availability Statement

The datasets presented in this study can be found in online repositories. The names of the repository/repositories and accession number(s) can be found below: https://www.ncbi.nlm.nih.gov/sra/PRJNA657679.

## Author Contributions

HZ performed most of the experiments, analyzed the data, and wrote the manuscript. LZ analyzed the transcriptomic data. YC, QX, and MW cultured heterotrophic seeds. MZ cultured photoautotrophic seeds. DH and QH designed the experiments and wrote the manuscript. All authors contributed to the final approval of the article.

## Conflict of Interest

The authors declare that the research was conducted in the absence of any commercial or financial relationships that could be construed as a potential conflict of interest.

## Footnotes

1 http://cegg.unige.ch/orthodb6
