# Supplementary material for: Trophic Transition Enhanced Biomass and Lipid Production of the Unicellular Green Alga Scenedesmus acuminatus
Source: Front Bioeng Biotechnol. 2021 May 21;9:638726. doi: 10.3389/fbioe.2021.638726 (PMC8176925; doi:10.3389/fbioe.2021.638726)
Supplement: Supplementary file 1 [file Data_Sheet_1.docx]

**Supplementary Materials**

**Trophic transition enhanced biomass and lipid production of the unicellular green alga *Scenedesmus acuminatus***

Hu Zhang^a, e^, Liang Zhao^a^, Yi Chen^a, e^, Mianmian Zhu^a, e^, Quan Xu^a, e^, Mingcan Wu^a^, Danxiang Han^a, c *^, Qiang Hu^a, b, c, d *^

^a^ Center for Microalgal Biotechnology and Biofuels, Institute of Hydrobiology, Chinese Academy of Sciences, Wuhan 430072, China

^b^ State Key Laboratory of Freshwater Ecology and Biotechnology, Institute of Hydrobiology, Chinese Academy of Sciences, Wuhan 430072, China;

^c^ Key Laboratory for Algal Biology, Institute of Hydrobiology, Chinese Academy of Sciences, Wuhan 430072, China

^d^ The Innovative Academy of Seed Design, Chinese Academy of Sciences, Beijing 100864, P. R. China

^e^ College of Life Sciences, University of Chinese Academy of Sciences, Beijing 100049, China

*Corresponding authors:

Danxiang Han ([danxianghan@ihb.ac.cn](mailto:danxianghan@ihb.ac.cn)), Qiang Hu ([huqiang@ihb.ac.cn](mailto:huqiang@ihb.ac.cn))


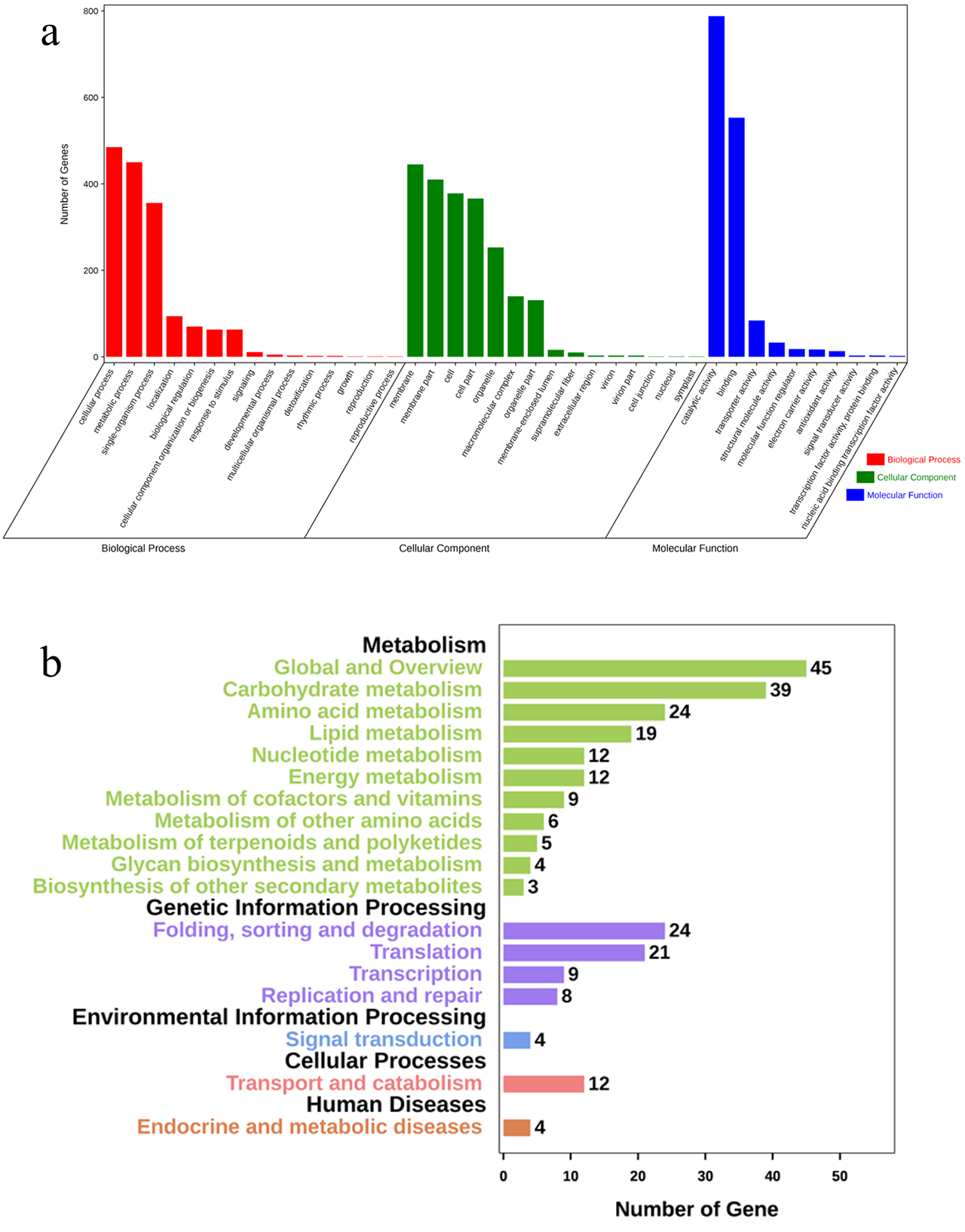


**Fig. S1** GO and KEGG annotation of differently expressed genes (DEGs) at 6 h of cultivation. a: GO enrichment analysis, b: KEGG enrichment pathway analysis.


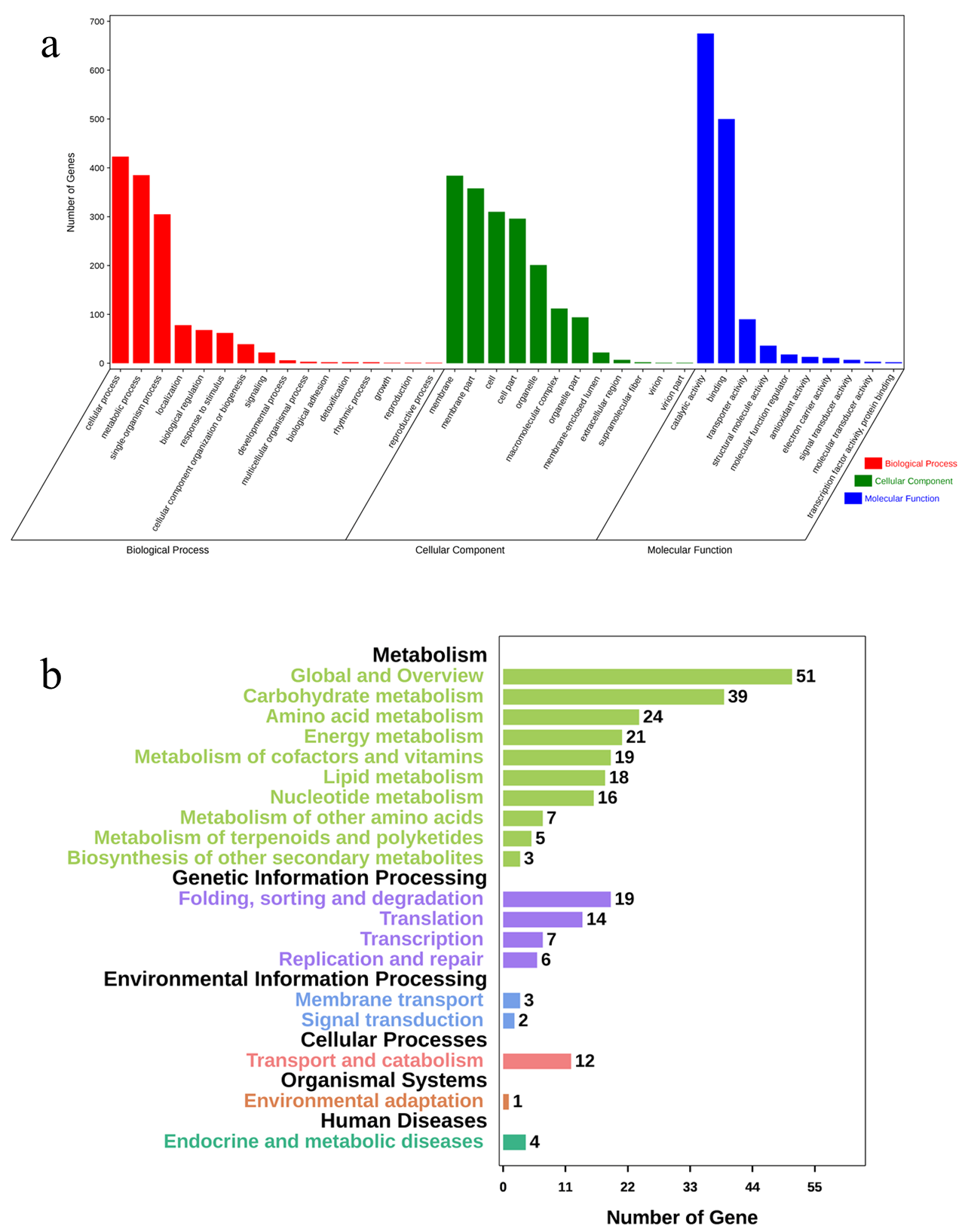


**Fig. S2** GO and KEGG annotation of DEGs at 12 h of cultivation. a: GO enrichment analysis, b: KEGG enrichment pathway analysis.


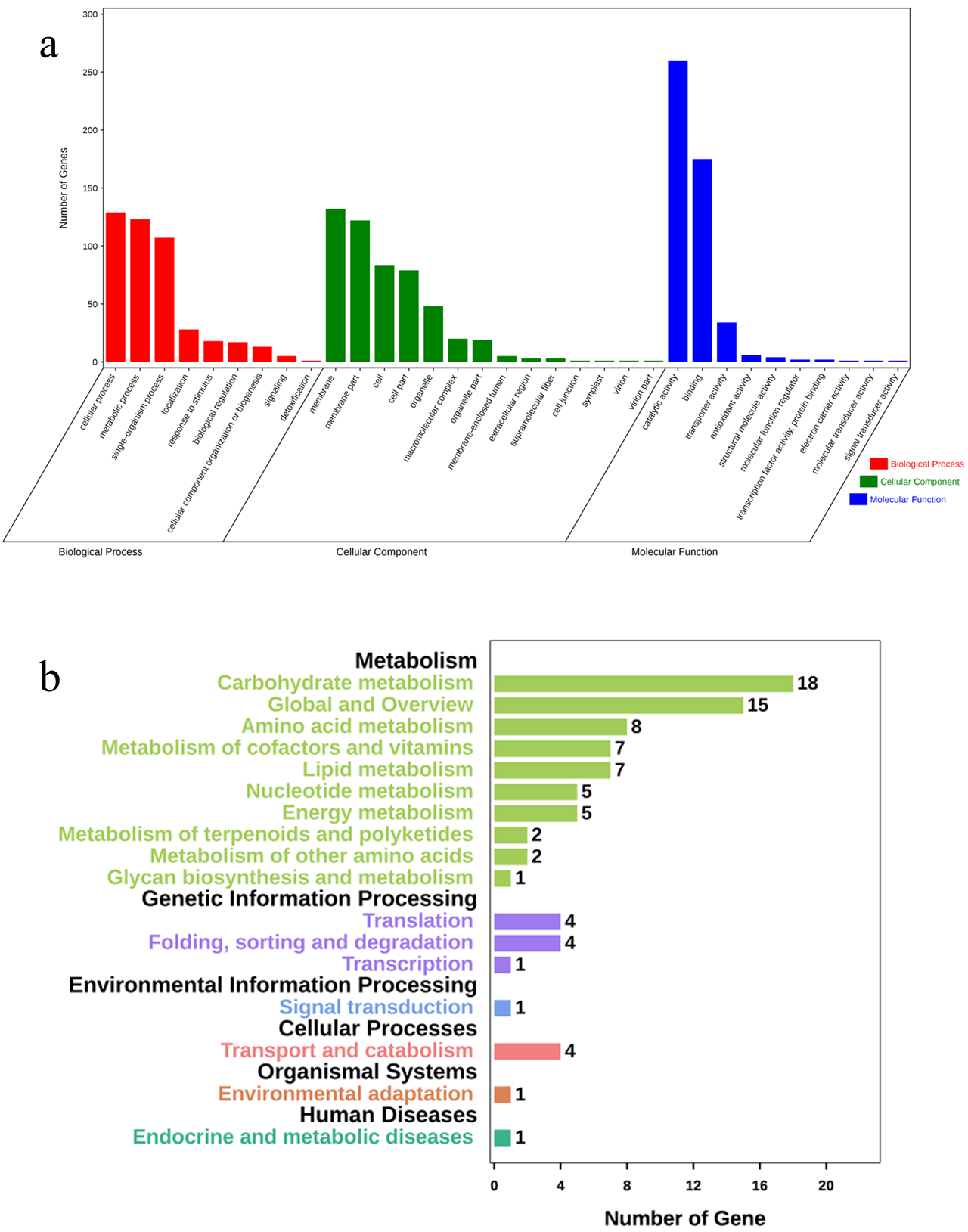


**Fig. S3** GO and KEGG annotation of DEGs at 24 h of cultivation. a: GO enrichment analysis, b: KEGG enrichment pathway analysis

**Table S1** Comparison of the fatty acid profiles of *S. acuminatus* between HC and PC culture during the cultivation.

| **Fatty acid** | **HC& Time (h)** | | | | **PC & Time (h)** | | | |
| --- | --- | --- | --- | --- | --- | --- | --- | --- |
| **profiles** | 0 | 48 | 96 | 144 | 0 | 48 | 96 | 144 |
| C16:0 | 23.14±2.38 | 27.31±1.93 | 25.03±1.96 | 26.95±1.75 | 17.11±0.46 | 27.87±1.46 | 25.57±2.09 | 25.82±2.09 |
| C16:1 | 5.05±0.14 | 2.04±0.00 | 1.79±0.03 | 1.59±0.00 | 2.44±0.07 | 1.16±0.09 | 1.67±0.08 | 1.76±0.09 |
| C16:2 | 3.23±0.00 | 2.92±0.11 | 3.31±0.03 | 3.20±0.07 | 3.36±0.00 | 2.70±0.09 | 3.32±0.11 | 3.45±0.11 |
| C16:3 | 4.63±0.14 | 2.31±0.06 | 2.11±0.03 | 1.82±0.07 | 3.44±0.15 | 2.27±0.13 | 2.14±0.20 | 2.09±0.20 |
| C16:4 | 5.33±0.14 | 2.20±0.00 | 1.06±0.03 | 0.98±0.07 | 3.67±0.23 | 2.48±0.30 | 0.85±0.35 | 0.81±0.01 |
| C18:0 | 1.68±0.00 | 2.75±0.06 | 3.63±0.06 | 2.99±0.09 | 2.60±0.15 | 3.42±0.09 | 3.45±0.11 | 3.71±0.11 |
| C18:1 | 31.84±2.38 | 35.30±2.81 | 42.44±2.61 | 41.83±2.01 | 35.83±0.92 | 34.72±2.44 | 42.42±2.11 | 42.93±2.11 |
| C18:2 | 11.64±1.16 | 9.25±0.06 | 8.65±0.15 | 8.48±0.14 | 12.30±0.31 | 10.10±0.56 | 8.75±0.77 | 8.32±0.77 |
| C18:3n3 | 13.46±0.28 | 15.91±0.83 | 11.99±0.79 | 12.15±0.40 | 19.25±0.61 | 15.28±1.24 | 11.83±0.81 | 11.11±0.81 |
| SFA | 24.82 | 30.06 | 28.66 | 29.94 | 19.71 | 31.29 | 29.02 | 29.53 |
| MUFA | 36.89 | 37.34 | 44.23 | 43.42 | 38.27 | 35.88 | 44.09 | 44.69 |
| PUFA | 38.29 | 32.59 | 27.12 | 26.63 | 42.02 | 32.83 | 26.89 | 25.78 |

Fatty acids were expressed as % of total fatty acid; SFA, saturated fatty acids; MUFA, monounsaturated fatty acids; PUFA, polyunsaturated fatty acids.

**Table S2** Comparison of chlorophyll content per cell dry weight and carotenoids content per cell dry weight of *S. acuminatus* between HC and PC culture during the cultivation.

|  | HC | | PC | |
| --- | --- | --- | --- | --- |
| Time  (h) | Chlorophyll content per cell dry weight (pg) | Carotenoids content per cell dry weight (pg) | Chlorophyll content per cell dry weight (pg) | Carotenoids content per cell dry weight (pg) |
| 0 | 11.01±0.48 | 3.65±0.03 | 25.03±0.19 | 7.25±0.11 |
| 24 | 7.41±0.49 | 2.57±0.09 | 9.74±0.05 | 4.18±0.06 |
| 48 | 2.96±0.11 | 1.43±0.06 | 3.62±0.11 | 2.42±0.06 |
| 72 | 1.81±0.04 | 1.05±0.03 | 1.58±0.04 | 1.81±0.11 |
| 96 | 1.23±0.02 | 0.85±0.02 | 1.11±0.03 | 1.56±0.14 |
| 120 | 0.90±0.03 | 0.73±0.01 | 0.87±0.01 | 1.27±0.06 |
| 144 | 0.69±0.04 | 0.65±0.01 | 0.77±0.01 | 1.11±0.05 |

**Table S3** Assembly statistics and assessment of transcriptome quality in *de novo* assembly of *S. acuminatus*

| **Assembly statistics** |  |
| --- | --- |
| No. of unigenes | 15,899 |
| Assembly size | 58,715,781 bp |
| N50 size | 1,193 bp |
| Average conting | 771.8 bp |
| GC content | 55.6% |
| **Quality assessment** |  |
| Total BUSCO groups search | 2,168 |
| Complete BUSCOs | 1,659 (76.5%) |
| Single copy | 1,134 (52.3%) |
| Duplicate copy | 525 (24.2%) |
| Fragmented BUSCOs | 230 (10.6%) |
| Missing BUSCOs | 279 (12.9%) |

**Table S4** Analysis of significant GO terms for Cellular Components of heterotrophically-grown *S. acuminatus* cells subjected to the high-light and N-limited conditions at 6, 12 and 24 hours of cultivation, respectively.

| **Time & genes** | **GO ID** | **Description** | **GeneRatio** | **BgRatio** | **FDR** |
| --- | --- | --- | --- | --- | --- |
| 6 h of cultivation with 730 DEGs in 4,558 AGs | 0009507 | chloroplast | 65 (8.90%) | 183 (4.01%) | 3.58E-11 |
|  | 0009536 | plastid | 66 (9.04%) | 189 (4.15%) | 5.95E-11 |
|  | 0044434 | chloroplast part | 39 (5.34%) | 104 (2.28%) | 6.55E-08 |
|  | 0009534 | chloroplast thylakoid | 20 (2.74%) | 57 (1.25%) | 3.16E-04 |
|  | 0009570 | chloroplast stroma | 14 (1.92%) | 35 (0.77%) | 5.54E-04 |
|  | 0009579 | thylakoid | 25 (3.42%) | 81 (1.78%) | 5.73E-04 |
| 12 h of cultivation with 631 DEGs in 4,558 AGs | 0009507 | chloroplast | 51 (8.08%) | 183 (4.01%) | 2.84E-07 |
|  | 0009536 | plastid | 52 (8.24%) | 189 (4.15%) | 3.38E-07 |
|  | 0009570 | chloroplast stroma | 15 (2.38%) | 35 (0.77%) | 2.47E-05 |
|  | 0044434 | chloroplast part | 29 (4.60%) | 104 (2.28%) | 1.11E-04 |
|  | 0044444 | cytoplasmic part | 155 (24.56%) | 876 (19.22%) | 2.07E-04 |
|  | 0005840 | ribosome | 44 (6.97%) | 191 (4.19%) | 3.07E-04 |
| 24 h of cultivation with 199 DEGs in 4,558 AGs | 0009501 | amyloplast | 4 (2.01%) | 5 (0.11%) | 1.70E-05 |
|  | 0016020 | membrane | 132 (66.33%) | 2,560 (56.16%) | 1.80E-03 |
|  | 0009507 | chloroplast | 17 (8.54%) | 183 (4.01%) | 2.36E-03 |
|  | 0044425 | membrane part | 122 (61.31%) | 2,376 (52.13%) | 4.81E-03 |
|  | 0005618 | cell wall | 3 (1.51%) | 14 (0.31%) | 2.09E-02 |

DEGs is differently expressed genes and AGs is annotated genes.

**Table S5** Changes in transcript expression of several genes related to photosynthetic carbon fixation and metabolism for the heterotrophically-grown *S. acuminatus* cells subjected to the high-light and N-limited conditions.

| **Gene_ID** | **KO_ID** | **Enzyme** | **Gene**  **_name** | **6h** | | **12h** | | **24h** | |
| --- | --- | --- | --- | --- | --- | --- | --- | --- | --- |
|  |  |  |  | **Log_2_FC** | **FDR** | **Log_2_FC** | **FDR** | **Log_2_FC** | **FDR** |
| c8501_c0_g1 | K01601 | ribulose-bisphosphate carboxylase large chain | rbcL | -1.950 | 5.71E-65 | -0.083 | 6.67E-01 | -1.651 | 6.90E-29 |
| c13567_c0_g1 | K01602 | ribulose-bisphosphate carboxylase small chain | rbcS | 0.313 | 5.63E-14 | -0.015 | 8.67E-01 | -2.939 | 0 |
| c14037_c0_g1 | K03841 | fructose-1,6-bisphosphatase I | PFB | -0.686 | 1.96E-48 | -0.852 | 1.03E-35 | -3.717 | 0.00E+00 |
| c18628_c1_g2 | K00927 | phosphoglycerate kinase | PGK | -0.398 | 4.46E-28 | 0.225 | 2.36E-03 | -5.108 | 0.00E+00 |
| c19271_c0_g1 | K01006 | pyruvate, orthophosphate dikinase | PPDK | 2.706 | 0.00E+00 | 1.669 | 4.21E-157 | 1.224 | 5.45E-87 |
| c13707_c0_g1 | K01595 | phosphoenolpyruvate carboxylase | PEPC | 0.245 | 1.48E-01 | 1.433 | 6.73E-33 | -1.408 | 7.87E-32 |
| c48143_c0_g1 | K01595 | phosphoenolpyruvate carboxylase | PEPC | 0.558 | 1.25E-04 | 1.771 | 1.65E-57 | -0.731 | 2.86E-06 |
| c15535_c0_g1 | K01610 | phosphoenolpyruvate carboxykinase | PEPCK | NA | NA | 2.593 | 8.31E-15 | NA | NA |
| c6221_c0_g1 | K00025 | malate dehydrogenase | MDH | -0.587 | 8.64E-12 | -2.686 | 1.87E-233 | 2.523 | 2.33E-185 |
| c17145_c0_g1 | K01783 | ribulose-phosphate 3-epimerase | RPE | -0.841 | 6.33E-85 | -0.314 | 2.17E-06 | -0.993 | 2.26E-35 |
| c2761_c0_g1 | K00615 | transketolase | TKT | 0.014 | 8.47E-01 | 0.348 | 1.45E-05 | -1.494 | 6.41E-58 |
| c12276_c0_g1 | K01807 | ribose 5-phosphate isomerase A | RPI | -0.605 | 9.48E-23 | -1.268 | 8.45E-57 | -2.112 | 1.42E-40 |

Log_2_FC is Log2 Fold Change. Positive value means upregulated and negative value means downregulated, NA means without detected gene.

**Table S6** Comparative transcriptomic analysis of central carbon metabolism for the heterotrophically-grown *S. acuminatus* cells subjected to the high-light and N-limited conditions.

| **Pathway** | **Gene_ID** | **KO_ID** | **Enzyme** | **Gene**  **_name** | **6h** | | **12h** | | **24h** | |
| --- | --- | --- | --- | --- | --- | --- | --- | --- | --- | --- |
|  |  |  |  |  | **Log_2_FC** | **FDR** | **Log_2_FC** | **FDR** | **Log_2_FC** | **FDR** |
| Glycolysis/  Gluconeog-enesis | c26733_c0_g1 | K00844 | hexokinase | HK | 2.965 | 5.44E-94 | 1.159 | 1.59E-26 | 0.292 | 6.40E-02 |
|  | c12829_c0_g1 | K01810 | glucose-6-phosphate isomerase | PGI | 0.645 | 5.69E-27 | 0.409 | 2.87E-08 | -1.896 | 2.40E-129 |
|  | c13173_c0_g1 | K00850 | 6-phosphofructokinase 1 | PFK-1 | 1.449 | 6.03E-70 | 1.373 | 1.70E-58 | -0.623 | 2.07E-07 |
|  | c14037_c0_g1 | K03841 | fructose-1,6-bisphosphatase I | PFB | -0.686 | 1.96E-48 | -0.852 | 1.03E-35 | -3.717 | 0.00E+00 |
|  | c18917_c0_g1 | K01623 | fructose-bisphosphate aldolase, class I | FBA | 0.459 | 1.97E-19 | 0.469 | 2.69E-03 | -2.414 | 1.01E-145 |
|  | c19278_c11_g1 | K01623 | fructose-bisphosphate aldolase, class I | FBA | -0.547 | 1.69E-05 | -0.654 | 1.73E-05 | -1.301 | 9.08E-08 |
|  | c41848_c0_g1 | K01623 | fructose-bisphosphate aldolase, class I | FBA | 0.275 | 1.90E-01 | -1.400 | 6.58E-19 | -2.912 | 2.14E-57 |
|  | c9005_c0_g1 | K01623 | fructose-bisphosphate aldolase, class I | FBA | 0.210 | 1.13E-01 | -0.629 | 4.32E-07 | -2.457 | 4.50E-43 |
|  | c19380_c3_g1 | K01803 | triosephosphate isomerase | TPI | 0.202 | 1.56E-04 | 0.191 | 7.59E-03 | -0.329 | 6.08E-04 |
|  | c19527_c6_g1 | K00134 | glyceraldehyde 3-phosphate dehydrogenase | GAP | -0.324 | 1.23E-03 | 0.110 | 3.70E-01 | 0.429 | 3.01E-76 |
|  | c18628_c1_g2 | K00927 | phosphoglycerate kinase | PGK | -0.398 | 4.46E-28 | 0.225 | 2.36E-03 | -5.108 | 0.00E+00 |
|  | c16257_c0_g1 | K01835 | phosphoglucomutase | PGM | -0.121 | 2.41E-03 | 0.018 | 8.59E-01 | -1.243 | 4.42E-67 |
|  | c35153_c0_g1 | K01835 | phosphoglucomutase | PGM | 1.668 | 7.71E-23 | 1.200 | 4.39E-21 | -1.061 | 2.30E-20 |
|  | c10776_c0_g1 | K01689 | enolase | ENO | 1.533 | 1.22E-30 | 1.701 | 1.20E-45 | -0.048 | 9.89E-01 |
|  | c13191_c0_g1 | K00873 | pyruvate kinase | PK | 1.348 | 4.28E-12 | 1.260 | 9.64E-09 | -1.887 | 3.81E-15 |
|  | c17436_c1_g1 | K00873 | pyruvate kinase | PK | 1.578 | 4.43E-12 | 1.909 | 2.71E-18 | -1.590 | 8.68E-10 |
|  | c18437_c1_g1 | K00873 | pyruvate kinase | PK | 2.219 | 1.20E-12 | 3.416 | 2.36E-52 | 0.206 | 5.11E-01 |
|  | c19271_c0_g1 | K01006 | pyruvate, orthophosphate dikinase | PPDK | 2.706 | 0.00E+00 | 1.669 | 4.21E-157 | 1.224 | 5.45E-87 |
|  | c19741_c0_g1 | K00161 | pyruvate dehydrogenase E1 component alpha subunit | PDH | 0.324 | 3.35E-05 | 0.790 | 1.72E-23 | -1.544 | 1.19E-81 |
|  | c2495_c0_g1 | K00162 | pyruvate dehydrogenase E1 component beta subunit | PDH | 0.841 | 7.50E-20 | 0.524 | 8.03E-08 | -1.839 | 3.55E-45 |
|  | c1232_c0_g1 | K00627 | pyruvate dehydrogenase E2 component | PDH | 0.480 | 1.12E-02 | 1.333 | 2.06E-15 | -1.043 | 5.93E-08 |
|  | c3191_c0_g1 | K00627 | pyruvate dehydrogenase E2 component | PDH | 0.463 | 1.05E-02 | 0.732 | 4.54E-06 | -0.555 | 2.40E-03 |
|  | c2366_c0_g1 | K01958 | pyruvate carboxylase | PC | 0.670 | 2.51E-19 | 0.519 | 3.74E-12 | 1.169 | 4.22E-41 |
|  | c13707_c0_g1 | K01595 | phosphoenolpyruvate carboxylase | PEPC | 0.245 | 1.48E-01 | 1.433 | 6.73E-33 | -1.408 | 7.87E-32 |

Table S6 Continued

| Glycolysis/  Gluconeog-  enesis | c48143_c0_g1 | K01595 | phosphoenolpyruvate carboxylase | PEPC | 0.558 | 1.25E-04 | 1.771 | 1.65E-57 | -0.731 | 2.86E-06 |
| --- | --- | --- | --- | --- | --- | --- | --- | --- | --- | --- |
|  | c15535_c0_g1 | K01610 | phosphoenolpyruvate carboxykinase | PEPCK | NA | NA | 2.593 | 8.31E-15 | NA | NA |
|  | c18498_c0_g2 | K01895 | Acetyl-CoA synthetase | ACS | 2.425 | 2.50E-182 | 2.465 | 4.33E-190 | -1.080 | 1.08E-22 |
| Citrate cycle  (TCA cycle) | c14761_c0_g1 | K01647 | citrate synthase | CS | 1.306 | 3.27E-53 | 1.729 | 1.21E-85 | 1.246 | 4.72E-31 |
|  | c19611_c4_g2 | K01647 | citrate synthase | CS | 0.716 | 1.80E-18 | 1.619 | 3.64E-136 | 0.181 | 4.81E-04 |
|  | c17663_c1_g1 | K01681 | aconitate hydratase | ACH | 0.512 | 4.07E-22 | 1.408 | 1.10E-77 | 0.556 | 7.79E-12 |
|  | c19288_c3_g4 | K00031 | isocitrate dehydrogenase | IDH | 0.407 | 2.56E-07 | -0.436 | 2.53E-07 | -2.181 | 2.46E-29 |
|  | c18077_c1_g2 | K00030 | isocitrate dehydrogenase (NAD+) | IDH | -0.353 | 2.04E-02 | 1.623 | 1.18E-37 | -1.978 | 1.42E-40 |
|  | c35142_c0_g1 | K00030 | isocitrate dehydrogenase (NAD+) | IDH | -0.402 | 5.34E-03 | 2.106 | 3.17E-78 | -1.978 | 1.04E-50 |
|  | c17217_c0_g1 | K00164 | 2-oxoglutarate dehydrogenase E1 component | OGD | 1.242 | 4.65E-125 | 1.233 | 2.39E-61 | 1.225 | 1.66E-64 |
|  | c16309_c0_g1 | K00658 | 2-oxoglutarate dehydrogenase E2 component | OGD | 0.835 | 1.06E-15 | 1.694 | 4.84E-47 | 1.223 | 5.31E-30 |
|  | c9281_c0_g1 | K01899 | succinyl-CoA synthetase alpha subunit | SCLA | 1.236 | 5.20E-52 | 1.441 | 1.92E-46 | 0.489 | 2.11E-06 |
|  | c14725_c0_g1 | K01900 | succinyl-CoA synthetase beta subunit | SCLA | 1.221 | 4.31E-55 | 1.958 | 1.65E-86 | 1.092 | 2.20E-36 |
|  | c15461_c0_g1 | K00234 | succinate dehydrogenase (ubiquinone) flavoprotein subunit | SDH | 1.658 | 4.60E-219 | 1.762 | 4.93E-122 | 1.061 | 1.12E-50 |
|  | c17615_c3_g1 | K00235 | succinate dehydrogenase (ubiquinone) iron-sulfur subunit | SDH | 1.329 | 2.27E-146 | 1.181 | 8.22E-68 | 0.770 | 1.36E-27 |
|  | c1086_c0_g1 | K00236 | succinate dehydrogenase (ubiquinone) cytochrome b560subunit | SDH | 1.087 | 7.62E-69 | 1.567 | 1.77E-72 | 0.879 | 3.00E-27 |
|  | c14176_c0_g1 | K01679 | fumarate hydratase, class II | FUM | 0.594 | 5.75E-08 | 1.034 | 3.54E-14 | 0.831 | 9.77E-14 |
|  | c6221_c0_g1 | K00025 | malate dehydrogenase | MDH | -0.587 | 8.64E-12 | -2.686 | 1.87E-233 | 2.523 | 2.33E-185 |
| Pentose phosphate pathway | c12829_c0_g1 | K01810 | glucose-6-phosphate isomerase | GPI | 0.645 | 5.69E-27 | 0.409 | 2.87E-08 | -1.896 | 2.40E-129 |
|  | c14573_c0_g1 | K00036 | glucose-6-phosphate 1-dehydrogenase | G6PD | 0.042 | 7.43E-01 | -1.220 | 3.06E-44 | -3.752 | 2.05E-265 |
|  | c15467_c0_g1 | K00036 | glucose-6-phosphate 1-dehydrogenase | G6PD | 0.762 | 6.38E-13 | 0.578 | 6.43E-05 | -0.384 | 6.43E-02 |
|  | c5724_c0_g1 | K01057 | 6-phosphogluconolactonase | PGL | 0.109 | 1.29E-01 | -0.110 | 1.91E-01 | 0.827 | 6.55E-18 |
|  | c18517_c0_g1 | K00033 | 6-phosphogluconate dehydrogenase | PGD | 0.374 | 2.74E-17 | -1.025 | 1.16E-64 | 1.027 | 2.02E-48 |
|  | c17145_c0_g1 | K01783 | ribulose-phosphate 3-epimerase | RPE | -0.841 | 6.33E-85 | -0.314 | 2.17E-06 | -0.993 | 2.26E-35 |
|  | c2761_c0_g1 | K00615 | transketolase | TKT | 0.014 | 8.47E-01 | 0.348 | 1.45E-05 | -1.494 | 6.41E-58 |
|  | c12276_c0_g1 | K01807 | ribose 5-phosphate isomerase A | RPI | -0.605 | 9.48E-23 | -1.268 | 8.45E-57 | -2.112 | 1.42E-40 |

Log_2_FC is Log2 Fold Change. Positive value means upregulated and negative value means downregulated, NA means without detected gene.

**Table S7** Comparative transcriptomic analysis of oxidative phosphorylation for the heterotrophically-grown *S. acuminatus* cells subjected to the high-light and N-limited conditions.

| **Gene_ID** | **KO_ID** | **Enzyme** | **Gene_name** | **6h** | | **12h** | | **24h** | |
| --- | --- | --- | --- | --- | --- | --- | --- | --- | --- |
|  |  |  |  | **Log_2_FC** | **FDR** | **Log_2_FC** | **FDR** | **Log_2_FC** | **FDR** |
| c15461_c0_g1 | K00234 | succinate dehydrogenase (ubiquinone) flavoprotein subunit | SDH | 1.653 | 4.60E-219 | 1.760 | 4.93E-122 | 1.110 | 1.12E-50 |
| c17615_c3_g1 | K00235 | succinate dehydrogenase (ubiquinone) iron-sulfur subunit | SDH | 1.324 | 2.27E-146 | 1.178 | 8.22E-68 | 0.819 | 1.36E-27 |
| c1086_c0_g1 | K00236 | succinate dehydrogenase (ubiquinone) cytochrome b560 subunit | SDH | 1.082 | 7.62E-69 | 1.564 | 1.77E-72 | 0.928 | 3.00E-27 |
| c19025_c8_g1 | K02257 | heme o synthase | COX10 | 1.169 | 6.42E-98 | 0.647 | 3.98E-15 | -0.125 | 2.69E-01 |
| c16651_c0_g1 | K02259 | cytochrome c oxidase assembly protein subunit 15 | COX15 | 0.402 | 2.27E-04 | 0.644 | 4.21E-157 | 1.253 | 6.76E-13 |
| c12514_c0_g1 | K02109 | F-type H+-transporting ATPase subunit b | ATPF0B | -0.216 | 1.69E-07 | -0.523 | 9.20E-16 | -3.084 | 0.00E+00 |
| c19297_c1_g1 | K02113 | F-type H+-transporting ATPase subunit delta | ATPF1D | -0.295 | 1.91E-12 | -0.329 | 4.95E-07 | -3.131 | 0.00E+00 |
| c19650_c0_g1 | K02115 | F-type H+-transporting ATPase subunit gamma | ATPF1G | 0.111 | 1.51E-02 | -0.493 | 2.05E-12 | -2.715 | 4.64E-186 |
| c996_c0_g1 | K02147 | V-type H+-transporting ATPase subunit B | ATPeV1B | 0.500 | 7.53E-15 | 1.307 | 1.34E-59 | -0.872 | 4.31E-20 |
| c17262_c1_g1 | K02148 | V-type H+-transporting ATPase subunit C | ATPeV1C | 1.228 | 4.01E-21 | 1.011 | 1.13E-11 | -1.308 | 2.51E-14 |
| c7787_c0_g1 | K02149 | V-type H+-transporting ATPase subunit D | ATPeV1D | 0.146 | 1.06E-01 | 1.013 | 3.37E-19 | -1.486 | 2.55E-31 |
| c20224_c0_g1 | K02150 | V-type H+-transporting ATPase subunit E | ATPeV1E | 1.178 | 2.06E-61 | 1.465 | 1.01E-66 | -0.484 | 8.96E-09 |

Log_2_FC is Log2 Fold Change. Positive value means upregulated and negative value means downregulated.

**Table S8** Changes in transcript expression of several genes related to antioxidant enzymes for the heterotrophically-grown *S. acuminatus* cells subjected to the high-light and N-limited conditions.

| **Gene_ID** | **Enzyme** | **6h** | | **12h** | | **24h** | |
| --- | --- | --- | --- | --- | --- | --- | --- |
|  |  | **Log_2_FC** | **FDR** | **Log_2_FC** | **FDR** | **Log_2_FC** | **FDR** |
| c19201_c0_g4 | superoxide dismutase 2 | -0.976 | 6.46E-30 | 1.213 | 1.75E-30 | -0.451 | 4.66E-06 |
| c16663_c0_g1 | iron-superoxide dismutase | -0.967 | 1.71E-60 | -0.864 | 3.16E-29 | -1.251 | 3.69E-42 |
| c19570_c2_g1 | catalase | -3.711 | 6.55E-179 | 2.220 | 1.37E-143 | -2.917 | 1.75E-101 |
| c5370_c0_g1 | catalase | 1.413 | 1.51E-125 | 0.693 | 5.62E-17 | 1.071 | 1.42E-29 |
| c8165_c0_g1 | glutathione peroxidase | -1.386 | 5.36E-16 | -2.411 | 1.51E-20 | -4.960 | 6.08E-32 |
| c757_c0_g1 | glutathione peroxidase | -3.932 | 9.76E-22 | -3.686 | 5.90E-24 | -2.457 | 9.57E-26 |

Log_2_FC is Log2 Fold Change. Positive value means upregulated and negative value means downregulated.

**Table S9** Comparative transcriptomic analysis of fatty acids and glycerolipid biosynthesis for the heterotrophically-grown *S. acuminatus* cells subjected to the high-light and N-limited conditions.

| **Category** | **Gene_ID** | **KO_ID** | **Enzyme** | **Gene_name** | **6 h** | | **12 h** | | **24 h** | |
| --- | --- | --- | --- | --- | --- | --- | --- | --- | --- | --- |
|  |  |  |  |  | **Log_2_FC** | **FDR** | **Log_2_FC** | **FDR** | **Log_2_FC** | **FDR** |
| Fatty acid biosynthesis | c48191_c0_g1 | K11262 | Acetyl-CoA carboxylase / biotin carboxylase | ACC | 1.557 | 1.30E-74 | 0.987 | 1.76E-32 | 2.714 | 4.75E-205 |
|  | c19681_c0_g1 | K01962 | Acetyl-CoA carboxylase carboxyl transferase subunit alpha | AccA | -1.893 | 8.55E-193 | -1.376 | 5.84E-64 | -2.339 | 3.33E-131 |
|  | c10403_c0_g1 | K02160 | Acetyl-CoA carboxylase biotin carboxyl carrier protein | AccB | -1.503 | 7.20E-69 | -0.874 | 7.88E-17 | -1.630 | 1.08E-54 |
|  | c12728_c0_g1 | K01961 | Acetyl-CoA carboxylase, biotin carboxylase subunit | AccC | -0.529 | 2.21E-11 | -0.063 | 5.56E-01 | -0.143 | 2.80E-01 |
|  | c19360_c4_g1 | K01963 | Acetyl-CoA carboxylase carboxyl transferase subunit beta | AccD | -2.133 | 2.48E-64 | -1.185 | 1.06E-16 | -2.048 | 1.19E-48 |
|  | c18664_c0_g1 | K00645 | Malonyl-CoA:ACP transferase | MAT | -1.123 | 1.80E-46 | 0.247 | 8.68E-03 | -3.164 | 0.00E+00 |
|  | c13660_c0_g1 | K00648 | 3-oxoacyl-ACP synthase III | KASIII | -1.913 | 1.81E-27 | -2.531 | 7.16E-31 | -3.799 | 6.84E-39 |
|  | c17273_c0_g1 | K09458 | 3-oxoacyl-ACP synthase II | KASII | -3.119 | 3.06E-88 | -3.269 | 2.66E-49 | -4.064 | 9.16E-53 |
|  | c41925_c0_g1 | K09458 | 3-oxoacyl-ACP synthase II | KASII | -0.798 | 6.66E-28 | -0.175 | 5.84E-02 | -2.284 | 3.87E-125 |
|  | c11347_c1_g1 | K00059 | Ketoxoacyl-ACP reductase | KAR | -0.512 | 1.95E-05 | -0.441 | 1.23E-03 | 0.396 | 4.65E-02 |
|  | c17550_c0_g2 | K00059 | Ketoxoacyl-ACP reductase | KAR | -1.874 | 2.50E-163 | -1.242 | 3.80E-37 | -2.635 | 4.42E-62 |
|  | c11232_c0_g1 | K02372 | 3-hydroxyacyl-ACP dehydratase | HD | -2.427 | 3.78E-29 | -2.314 | 6.18E-26 | -3.484 | 3.38E-36 |
|  | c12357_c0_g1 | K00208 | Enoyl-ACP reductase I | ENR | -1.990 | 3.34E-260 | -0.351 | 1.22E-05 | -2.470 | 2.93E-254 |
|  | c12435_c0_g1 | K10782 | Fatty acyl-ACP thioesterase A | FATA | -1.930 | 2.51E-11 | -2.218 | 3.29E-15 | -0.858 | 5.44E-04 |
|  | c19204_c0_g1 | K03921 | Acyl-ACP desaturase | DESA | -1.793 | 7.43E-227 | -1.035 | 2.35E-39 | -1.300 | 1.99E-58 |
| Fatty acid  elongation | c19355_c5_g1 | K01897 | Long-chain acyl-CoA synthetase | LC-FACS | 0.146 | 1.93E-02 | -0.392 | 2.39E-07 | -0.885 | 6.80E-23 |
|  | c20070_c0_g1 | K01897 | Long-chain acyl-CoA synthetase | LC-FACS | 1.501 | 8.73E-54 | 0.874 | 5.90E-21 | 0.932 | 2.43E-15 |
|  | c2595_c0_g1 | K07511 | Enoyl-CoA hydratase | ECHS | 0.714 | 7.88E-05 | -0.288 | 2.29E-01 | -0.820 | 1.88E-05 |
|  | c13252_c0_g1 | K07512 | Trans-2-enoyl-CoA reductase | ECR | -0.362 | 8.79E-09 | -0.147 | 1.29E-01 | -0.483 | 5.13E-07 |
|  | c16743_c0_g1 | K01074 | Palmitoyl-protein thioesterase | PPT | 0.406 | 1.89E-11 | 0.541 | 2.64E-14 | -0.173 | 3.35E-02 |
|  | c19313_c4_g11 | K15397 | 3-ketoacyl-CoA synthase | KCS | 0.639 | 2.16E-03 | 0.208 | 4.70E-02 | -1.191 | 2.95E-06 |

Table S9 Continued

| Fatty acid  elongation | c19313_c4_g2 | K15397 | 3-ketoacyl-CoA synthase | KCS | 3.041 | 1.50E-119 | 0.114 | 1.76E-01 | 0.627 | 1.72E-06 |
| --- | --- | --- | --- | --- | --- | --- | --- | --- | --- | --- |
|  | c19313_c4_g7 | K15397 | 3-ketoacyl-CoA synthase | KCS | 1.377 | 1.19E-20 | -0.179 | 8.23E-02 | 0.560 | 7.28E-04 |
|  | c19313_c4_g8 | K15397 | 3-ketoacyl-CoA synthase | KCS | -0.099 | 3.34E-01 | 0.624 | 8.50E-18 | 0.027 | 7.97E-01 |
|  | c19313_c4_g9 | K15397 | 3-ketoacyl-CoA synthase | KCS | 0.435 | 2.43E-02 | -0.955 | 6.54E-15 | -1.492 | 5.16E-14 |
|  | c15742_c0_g1 | K10251 | Very-long-chain 3-oxoacyl-CoA reductase | VLCOCR | 0.172 | 2.98E-01 | 0.659 | 1.55E-04 | 0.742 | 1.32E-04 |
|  | c16941_c1_g2 | K10703 | Very-long-chain (3R)-3-hydroxyacyl-CoA dehydratase | VLCHCD | 1.140 | 5.12E-47 | -0.834 | 1.56E-17 | -1.333 | 7.89E-35 |
|  | c36371_c0_g1 | K10258 | Very-long-chain enoyl-CoA reductase | VLCECR | 1.073 | 7.51E-64 | -0.428 | 3.22E-07 | 0.004 | 9.69E-01 |
| Glycerolipid biosynthesis | c16592_c0_g1 | K13506 | Glycerol-3-phosphate O-acyltransferase | GPAT | 0.037 | 7.88E-01 | -0.170 | 3.47E-01 | -1.173 | 1.13E-08 |
|  | c19509_c0_g2 | K00630 | Glycerol-3-phosphate O-acyltransferase | GPAT | 0.001 | 9.96E-01 | -1.121 | 3.18E-33 | -1.750 | 8.89E-32 |
|  | c17419_c0_g1 | K13519 | Lysophospholipid acyltransferase | LPAAT | -0.415 | 2.55E-02 | -0.555 | 1.30E-02 | -0.463 | 1.65E-01 |
|  | c19421_c6_g5 | K00901 | Diacylglycerol kinase | DAGK | 0.237 | 1.74E-02 | 0.209 | 1.68E-02 | 0.709 | 5.62E-13 |
|  | c41495_c0_g1 | K00901 | Diacylglycerol kinase | DAGK | 0.251 | 2.42E-01 | 0.151 | 3.88E-01 | 0.511 | 1.36E-02 |
|  | c16441_c0_g1 | K22848 | Diacylglycerol acyltransferase 2 | DGAT2 | 1.120 | 8.33E-07 | 1.400 | 1.31E-05 | 1.763 | 6.02E-16 |
|  | c17093_c0_g1 | K22848 | Diacylglycerol acyltransferase 2 | DGAT2 | -0.466 | 1.18E-15 | -0.604 | 2.58E-17 | 0.521 | 1.02E-07 |
|  | c17825_c1_g1 | K22848 | Diacylglycerol acyltransferase 2 | DGAT2 | -0.631 | 9.54E-04 | -0.746 | 4.76E-04 | -0.565 | 9.38E-02 |
|  | c7026_c0_g1 | K22848 | Diacylglycerol acyltransferase 2 | DGAT2 | -0.930 | 9.22E-24 | -0.398 | 8.74E-03 | 1.722 | 3.64E-15 |
|  | c11354_c0_g1 | K00679 | Phospholipid:diacylglycerol acyltransferase | PDAT | -0.653 | 1.87E-04 | 0.694 | 6.80E-05 | 0.617 | 5.28E-03 |
|  | c55116_c0_g1 | K06118 | UDP-sulfoquinovose synthase | SQD1 | -0.758 | 9.47E-46 | -0.470 | 9.96E-10 | -2.041 | 3.62E-121 |
|  | c14243_c0_g1 | K06119 | Sulfoquinovosyltransferase | SQD2 | 0.326 | 2.73E-07 | 0.468 | 3.22E-09 | -0.391 | 1.06E-05 |
|  | c18633_c0_g2 | K09480 | Digalactosyldiacylglycerol synthase | DGD | 0.554 | 8.53E-06 | 0.502 | 2.42E-07 | 0.542 | 2.36E-09 |
|  | c19355_c4_g2 | K00006 | Glycerol-3-phosphate dehydrogenase | G3PDH | 1.418 | 1.29E-28 | 2.565 | 1.16E-94 | -0.669 | 4.99E-09 |
|  | c34159_c0_g1 | K00111 | Glycerol-3-phosphate dehydrogenase | G3PDH | 0.644 | 3.90E-06 | 0.213 | 1.39E-01 | -0.447 | 4.84E-02 |
|  | c17285_c1_g1 | K01080 | Phosphatidate phosphatase | PAP | 0.089 | 6.46E-01 | 0.049 | 8.22E-01 | 0.669 | 4.69E-02 |
|  | c18937_c5_g1 | K01115 | Phospholipase | PLA | 0.937 | 2.08E-09 | 0.935 | 2.81E-12 | -0.025 | 9.03E-01 |
|  | c16347_c0_g1 | K13510 | Lysophosphatidylcholine acyltransferase | LPCAT | -1.026 | 4.88E-30 | -0.382 | 2.37E-04 | -0.217 | 1.96E-01 |
|  | c16896_c0_g1 | K06130 | Lysophospholipase II | LPLA | -0.440 | 1.66E-07 | -0.247 | 4.16E-02 | -1.008 | 2.40E-15 |

Log_2_FC is Log2 Fold Change. Positive value means upregulated and negative value means downregulated

**Table S10** Comparative transcriptomic analysis of starch metabolism for the heterotrophically-grown *S. acuminatus* cells subjected to the high-light and N-limited conditions.

| **Gene_ID** | **KO_ID** | **Enzyme** | **Gene_name** | **6 h** | | **12 h** | | **24 h** | |
| --- | --- | --- | --- | --- | --- | --- | --- | --- | --- |
|  |  |  |  | **Log_2_FC** | **FDR** | **Log_2_FC** | **FDR** | **Log_2_FC** | **FDR** |
| c12829_c0_g1 | K01810 | Phosphoglucose isomerase | PGI | 0.641 | 5.69E-27 | 0.406 | 2.87E-08 | -1.847 | 2.4E-129 |
| c16257_c0_g1 | K01835 | Phosphoglucomutase | PGM | -0.126 | 0.002408 | 0.016 | 0.859205 | -1.194 | 4.42E-67 |
| c35153_c0_g1 | K01835 | Phosphoglucomutase | PGM | 1.663 | 7.71E-23 | 1.198 | 4.39E-21 | -1.012 | 2.3E-20 |
| c12816_c0_g1 | K00703 | Starch synthase | SS | 0.604 | 1.88E-07 | 0.326 | 0.001392 | -2.912 | 3.13E-84 |
| c14371_c1_g1 | K00703 | Starch synthase | SS | -0.296 | 0.101717 | -0.914 | 1.09E-11 | -3.942 | 1.7E-154 |
| c16245_c0_g1 | K00703 | Starch synthase | SS | 0.1 | 0.389697 | -0.123 | 0.273808 | -0.031 | 0.757328 |
| c18188_c2_g1 | K00703 | Starch synthase | SS | 0.771 | 4.22E-06 | 0.338 | 0.006329 | -2.67 | 3.04E-61 |
| c18847_c3_g1 | K00703 | Starch synthase | SS | -1.092 | 1.16E-11 | -1.512 | 1.23E-19 | -3.668 | 5.26E-81 |
| c19380_c3_g2 | K00703 | Starch synthase | SS | -0.173 | 0.185493 | -0.638 | 3.2E-09 | 0.24 | 0.028385 |
| c19621_c3_g3 | K13679 | Granule-bound starch synthase | GBSS | -1.251 | 3.6E-215 | -1.507 | 2.9E-118 | -3.014 | 0 |
| c17672_c0_g1 | K01176 | Alpha-amylase | AMY | 0.258 | 0.000298 | -0.183 | 0.011608 | -0.298 | 0.002814 |
| c18896_c0_g2 | K01176 | Alpha-amylase | AMY | -1.973 | 8.71E-46 | 0.445 | 0.000885 | -0.694 | 0.000309 |
| c18352_c1_g1 | K01177 | Beta-amylase | BMY | -0.642 | 1.67E-06 | -0.245 | 0.048442 | -0.639 | 0.000165 |
| c18575_c12_g1 | K01177 | Beta-amylase | BMY | 0.655 | 0.001713 | 0.398 | 0.002235 | -0.577 | 0.000183 |
| c41260_c0_g1 | K01177 | Beta-amylase | BMY | 1.185 | 6.94E-07 | 0.464 | 0.001051 | -0.344 | 0.022536 |
| c10711_c1_g1 | K01214 | Isoamylase | ISA | -0.726 | 2.79E-09 | -0.952 | 7.84E-19 | -3.444 | 3.2E-160 |
| c18250_c0_g1 | K01214 | Isoamylase | ISA | 0.51 | 3.01E-05 | 0.378 | 0.008776 | -1.383 | 6.63E-09 |
| c9729_c0_g1 | K01214 | Isoamylase | ISA | 0.1 | 0.447502 | 0.264 | 0.081181 | -2.598 | 1.92E-42 |

Log_2_FC is Log2 Fold Change. Positive value means upregulated and negative value means downregulated

**Table S11** Comparative transcriptomic analysis of nitrogen metabolism for the heterotrophically-grown *S. acuminatus* cells subjected to the high-light and N-limited conditions.

| **Gene_ID** | **KO_ID** | **Enzyme** | **Gene_name** | **6 h** | | **12 h** | | **24 h** | |
| --- | --- | --- | --- | --- | --- | --- | --- | --- | --- |
|  |  |  |  | **Log_2_FC** | **FDR** | **Log_2_FC** | **FDR** | **Log_2_FC** | **FDR** |
| c1778_c0_g1 | K02575 | Nitrate/nitrite transporter | NRT | -3.505 | 5.24E-94 | -1.602 | 1.01E-22 | 7.038 | 0 |
| c3292_c0_g1 | K10534 | Nitrate reductase (NAD(P)H) | NR | -0.426 | 7.33E-05 | -3.184 | 2.68E-169 | 4.226 | 0 |
| c13261_c0_g1 | K00363 | Ferredoxin-nitrite reductase | FNR | -2.345 | 2.36206E-57 | -2.595 | 2.4147E-101 | 6.929 | 3E-273 |
| c18130_c4_g2 | K00261 | Glutamate dehydrogenase (NAD(P)+) | GD | 0.963 | 1.23E-47 | 1.266 | 1.77E-61 | 0.285 | 0.009 |
| c15013_c0_g1 | K01915 | Glutamine synthetase | GS | 0.627 | 4.43896E-19 | 3.504 | 0 | 1.046 | 2E-45 |
| c19096_c0_g1 | K00264 | Glutamate synthase (NADH) | GSN | 2.193 | 6.7269E-280 | 3.585602447 | 0 | 1.347 | ###### |
| c11870_c0_g1 | K00284 | Glutamate synthase (ferredoxin) | GSF | -0.641 | 1.02323E-05 | -0.793 | 1.16872E-12 | -2.994 | 2E-74 |
| c19146_c0_g1 | K01725 | Cyanate lyase | CL | -1.275 | 3.04E-29 | -0.997 | 4.90434E-16 | 0.669 | 5E-07 |
| c18834_c2_g1 | K01674 | Carbonic anhydrase | CA | -0.978 | 2.81081E-14 | -1.568 | 1.50969E-23 | -1.079 | 6E-06 |

Log_2_FC is Log2 Fold Change. Positive value means upregulated and negative value means downregulated.
